# Sigma-1-targeting multimodal compound HBK-15 reverses memory deficits and restores hippocampal plasticity under NMDA hypofunction

**DOI:** 10.1016/j.neurot.2025.e00774

**Published:** 2025-11-03

**Authors:** Kinga Sałaciak, Klaudia Lustyk, Angelika Jagielska, Małgorzata Szafarz, Sara Inteiro-Oliveira, Maria José Diógenes, Sara Xapelli, Paulina Schnur, Lucy Morton, Erin Moran, Jacques Ferreira, Shuzo Sakata, Lucie Crouzier, Johann Meunier, Benjamin Delprat, Tangui Maurice, Karolina Pytka

**Affiliations:** a Laboratory of Experimental Neuropharmacology, Department of Pharmacodynamics, Faculty of Pharmacy, Jagiellonian University Medical College, 9 Medyczna St, 30-688 Krakow, Poland; b Doctoral School of Medical and Health Sciences, Jagiellonian University Medical College, 16 Św. Łazarza St, 31-530 Krakow, Poland; c Department of Pharmacokinetics and Physical Pharmacy, Faculty of Pharmacy, Jagiellonian University Medical College, 9 Medyczna St, 30-688 Krakow, Poland; d Centro Cardiovascular da Universidade de Lisboa (CCUL@RISE), Faculdade de Medicina, Universidade de Lisboa, Lisboa, Portugal; e GIMM - Gulbenkian Institute for Molecular Medicine, Lisboa, Portugal; f Strathclyde Institute of Pharmacy and Biomedical Sciences, University of Strathclyde, 161 Cathedral St, Glasgow G4 0RE, UK; g MMDN, Univ Montpellier, EPHE, INSERM, Montpellier, France

**Keywords:** Sigma-1 receptor, NMDA receptor hypofunction, Cognitive impairments, Depression, Schizophrenia

## Abstract

Memory impairment is among the most disabling features of depression and schizophrenia, yet remains largely untreated by available pharmacotherapies. NMDA receptor hypofunction is strongly implicated in these deficits, while sigma-1 receptors, by stabilizing calcium signaling and supporting glutamatergic plasticity, have emerged as a promising therapeutic target. HBK-15, a methoxyphenylpiperazine derivative with a multimodal receptor profile, had previously shown preliminary anti-amnesic activity in rodents, prompting us to test its efficacy under NMDA receptor hypofunction. We therefore investigated whether HBK-15 engages sigma-1 receptors and restores memory in a mouse model of MK-801-induced impairment. HBK-15 bound sigma-1 receptors with high affinity and showed functional agonist activity in the BiP assay. Behaviorally, HBK-15 reversed MK-801-induced recognition and spatial memory deficits across acquisition and retrieval phases, similar to encoding and delayed recall in clinical settings. In contrast, vortioxetine and lurasidone showed only limited benefits, highlighting the broader effectiveness of HBK-15. Its ability to reverse memory impairment depended on sigma-1 receptor activity, emphasizing this pathway as a key therapeutic target. Mechanistically, HBK-15 increased hippocampal glutamatergic and cholinergic signaling under NMDA blockade, restored long-term potentiation, and improved disrupted theta-gamma coupling, a network correlate of hippocampal memory function. These findings offer experimental evidence that HBK-15 activates sigma-1 receptors to enhance hippocampal plasticity at both synaptic and network levels and to improve memory under NMDA hypofunction. Taken together, our results highlight sigma-1-based strategies as a tractable avenue for developing treatments targeting cognitive symptoms in depression and schizophrenia.

## Introduction

Cognitive impairments are central to the course of depression and schizophrenia, predicting disability, relapse, and poor functional recovery [1,2]. Yet current pharmacotherapies focus almost exclusively on mood or psychotic symptoms. Among the most disabling are memory disturbances, which remain largely untreated [3]. Developing interventions that directly restore memory thus represents an urgent unmet therapeutic need in neuropsychiatric disorders.

Glutamatergic dysfunction, especially N-methyl-D-aspartate (NMDA) receptor hypofunction, is strongly implicated in the cognitive disturbances of depression and schizophrenia [4]. Converging post-mortem, imaging, and electrophysiological evidence links reduced NMDA receptor–mediated signaling, particularly within cortical interneuron networks, to disinhibition, altered gamma oscillations, and impaired plasticity in these disorders [[5], [6], [7], [8], [9], [10]]. This pattern of cortical NMDA receptor hypofunction, which disrupts network synchrony and impairs higher-order cognitive processing, represents a core pathophysiological feature underlying cognitive deficits in depression and schizophrenia and differs fundamentally from the glutamatergic overactivation and excitotoxicity observed in neurodegenerative conditions such as Alzheimer's or Huntington's diseases [11]. As disrupted NMDA signaling impairs synaptic plasticity and memory processes [12,13], modulatory systems capable of stabilizing glutamatergic function are of particular interest. The sigma-1 receptor has emerged as a key candidate, acting as a ligand-regulated chaperone at mitochondria-associated membranes that modulates calcium signaling, boosts NMDA receptor-mediated neuronal activity [14], supports long-term potentiation, and regulates neurotransmitter release [15]. Preclinical studies consistently reveal that sigma-1 receptor activation yields anti-amnesic effects, particularly in models of NMDA receptor hypofunction [16] underscoring its translational potential as a therapeutic target (reviewed in Ref. [17]).

HBK-15, a methoxyphenylpiperazine derivative with a multimodal receptor profile [[18], [19], [20], [21], [22]], shows the highest affinity for the 5-HT_1A_ receptor and lower affinity for 5-HT_7_, D_2_, and α_1_ receptors [[18], [19], [20], [21], [22], [23]]. The compound displayed functional selectivity at 5-HT_1A_ and 5-HT_7_ receptors, partially activating ERK1/2 phosphorylation and cAMP production, respectively [24]. HBK-15 has previously demonstrated rapid antidepressant- and anxiolytic-like effects and showed initial evidence of anti-amnesic activity in cholinergic dysfunction-induced amnesia [20,21,25]. Notably, despite its antagonistic activity at α_1_ and D_2_ receptors, the compound did not lower blood pressure, induce antipsychotic-like effects, or produce catalepsy [23,26]. Importantly, HBK-15 was well tolerated and brain-penetrant in animal studies, further supporting its translational potential [26,27]. Although the pharmacological profile of HBK-15 is well defined at serotonergic, dopaminergic, and adrenergic targets, its potential interactions with other modulatory systems remain to be fully elucidated. Based on its chemical structure and broad receptor interactions, we hypothesized that HBK-15 may also engage the sigma-1 receptor, a mechanism potentially underlying its anti-amnesic efficacy. However, whether HBK-15 can counteract memory disturbances driven by glutamatergic dysfunction has not yet been established.

Here, we tested if HBK-15 reverses recognition and spatial memory deficits induced by NMDA blockade. Given that both acquisition/encoding and retrieval failures contribute to cognitive disability in depression and schizophrenia [28,29], we specifically examined these phases in our study. To probe mechanisms, we profiled HBK-15's receptor interactions, assessed functional sigma-1 activity, and examined hippocampal correlates of plasticity across neurotransmitter signaling, long-term potentiation, and network oscillations. This integrated approach allowed us to test the hypothesis that HBK-15 restores memory via sigma-1 receptor activation and stabilizes hippocampal plasticity at multiple levels.

## Materials and Methods

Detailed methods description can be found in the Supplementary materials.

### Animals

Depending on the experiment, we used naïve adult male BALB/c (Mossakowski Medical Research Institute, Polish Academy of Sciences, or Charles River Laboratories (Barcelona, Spain or Edinburgh, UK)) or C57BL/6J (Mossakowski Medical Research Institute, Polish Academy of Sciences) weighing 24 ​± ​2 ​g (around 8-10 weeks old). Any variation in group sizes resulted from experimental losses, violations of predetermined exclusion criteria, or exclusions based on outlier identification using the ROUT method. All experiments were performed following the European (2012/707/EU) and Polish, Portuguese, or the United Kingdom Animals (Scientific Procedures) Act of 1986, Home Office regulations (PP0688944).

### Drugs

1-[(2-chloro-6-methylphenoxy)ethoxyethyl]-4-(2-methoxyphenyl)piperazine hydrochloride (HBK-15, Fig. S1) was synthesized in the Department of Technology and Biotechnology of Drugs, Faculty of Pharmacy, Jagiellonian University Medical College, according to the methodology described earlier [22]. HBK-15, MK-801 (dizocilpine; Sigma-Aldrich, Germany), BD 1047, vortioxetine, and lurasidone (STI, Poland) were dissolved in saline and administered intraperitoneally (*ip*). Control groups received saline. Vortioxetine, an antidepressant with multimodal serotonergic activity, and lurasidone, an atypical antipsychotic targeting serotonergic and dopaminergic receptors, were used as reference compounds to compare HBK-15 with existing treatments known for their cognitive-enhancing effects. The doses of the studied compounds, MK-801 and BD 1047, for experiments were based on earlier studies and literature data [18,30].

For electrophysiological experiments, HBK-15 was supplied in a 1.0 ​mg/ml stock solution in saline and added to the slices in 1 ​μg/ml diluted in the artificial cerebral spinal fluid (aCSF). MK-801 was dissolved in a 10 ​mM stock solution in saline and added to the slices in a 10 ​μM dilution in aCSF.

### *In vitro* binding assays

Binding studies were performed commercially in Eurofins Laboratories using testing procedures described elsewhere (Table S1): sigma-1 and sigma-2 receptors [31], kainate (KA), α-amino-3-hydroxy-5-methyl-4-isoxazolepropionic acid (AMPA) [32], NMDA (Glu) [33], glycine (Gly) [34], MK-801 [35], and polyamine(PA) sites [36], 5-HT_3_ [37], N-type calcium channels Cav2.2.; (*ω*-conotoxin) [38], N-type calcium channels (Cav2.2.; gabapentin) [39], L-type calcium channels (Cav1.2.; phenylalkylamine) [40], mGluR1 [41], mGluR2 [42] and mGluR5 [41]. The results are presented as the inhibition of control-specific binding in the presence of HBK-15. For inhibition values above 60 ​%, the negative logarithm of the inhibition constant (*p*Ki) was calculated.

### S1R/BiP dissociation assay

**Fig. 1:**
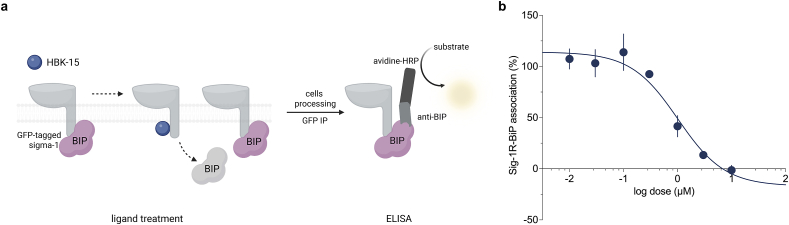
**HBK-15 showed agonistic properties toward sigma-1 receptors. a**: Schematic overview of the BiP-dissociation assay for sigma-1 receptor agonistic activity of HBK-15. Cells expressing GFP-tagged sigma-1 receptors were treated with HBK-15, cross-linked, and lysed. BiP–receptor complexes were isolated by GFP immunoprecipitation (GFP IP) using GFP-trap agarose and quantified by ELISA with an anti-BiP antibody and horseradish peroxidase (HRP)-based detection (created with BioRender.com). **b**: Dose-response curve of the effect of HBK-15 in S1R/BiP dissociation assay.

The assay followed the method by Hayashi & Su [43] with an optimized protocol [44,45] (Fig. 1a). CHO cells overexpressing GFP-tagged S1R (gift from Dr Tsung-Ping Su and Dr Yuko Yasui, IRP, NIDA/NIH, Baltimore, MD, USA) were cultured in DMEM/Glutamax with 10 ​% heat-inactivated FBS, plated in 12-well plates, and treated with the test drug for 30 ​min at 37 ​°C. The reaction was stopped by replacing the medium with PBS, and cells were cross-linked with dithiobis succinimidyl propionate. After stopping the reaction with Tris/HCl, cells were lysed, and the supernatant was incubated with GFP-trap agarose overnight at 4 ​°C. The pellet was then washed and analyzed using a Heat Shock 70 ​KDa Protein 5 ELISA assay.

### Behavioral tests

Behavioral tests were carried out as previously described (object recognition test [46,47]; Morris water maze [48,49]) and detailed in the Supplementary materials.

#### Object recognition test

*Familiarization session*: mice were placed individually in the open-field with two identical objects and left there until they reached the 20-s criterion of total exploration, but no longer than 10 ​min. Animals that did not meet this criterion were excluded from further studies.

*Test phase*: mice were placed again in the open-field, but this time one of the objects was replaced with a new one. Mice were again left until they reached the 20-s criterion of total exploration, but no longer than 10 ​min. To assess animals' performance in the object recognition test, the means of the novel object exploration time were compared with the chance level (10 ​s, equal exploration of the objects).

**Fig. 2:**
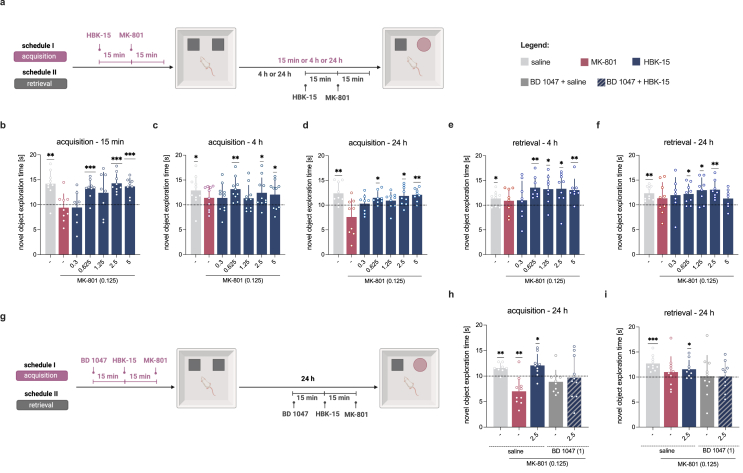
**HBK-15 rescued MK-801-induced recognition memory deficits in a sigma-1-dependent manner. a:** Experimental scheme: HBK-15 and MK-801 were administered intraperitoneally (*ip*) before the familiarization phase to assess encoding, and before the test phase to evaluate retrieval (created with BioRender.com). **b,c,d:** The effect of HBK-15 on the novel object exploration time 15 ​min (**b**), 4 ​h (**c**), and 24 ​h ​(**d**) post-familiarization phase during the encoding stage. **e,f**: The influence of HBK-15 on the novel object exploration time 4 ​h (**e**) and 24 ​h (**f**) post-familiarization phase at the stage of memory retrieval. The data are presented as means ​± ​SD. Statistical analysis: one-sample *t*-test, ∗*p* ​< ​0.05, ∗∗*p* ​< ​0.01, ∗∗∗*p* ​< ​0.001 *vs* chance level ​= ​10s; acquisition: 15 ​min–0.625 ​mg/kg: *t*(8) ​= ​5.253, *p* ​< ​0.001; 2.5 ​mg/kg: *t*(8) ​= ​6.766, *p* ​< ​0.001; 5 ​mg/kg: *t*(7) ​= ​5.955, *p* ​< ​0.001; 4 ​h–0.625 ​mg/kg: *t*(9) ​= ​4.345, *p* ​< ​0.01; 2.5 ​mg/kg: *t*(9) ​= ​2.561, *p* ​< ​0.05; 5 ​mg/kg: *t*(9) ​= ​2.275, *p* ​< ​0.05; 24 ​h–0.625 ​mg/kg: *t*(7) ​= ​3.251, *p* ​< ​0.05; 2.5 ​mg/kg: *t*(8) ​= ​2.954, *p* ​< ​0.05; 5 ​mg/kg: *t*(7) ​= ​4.548, *p* ​< ​0.01; retrieval: 4 ​h–0.625 ​mg/kg: *t*(7) ​= ​4.714, *p* ​< ​0.01; 1.25 ​mg/kg: *t*(7) ​= ​3.128, *p* ​< ​0.05; 2.5 ​mg/kg: *t*(8) ​= ​3.014, *p* ​< ​0.05; 5 ​mg/kg: *t*(7) ​= ​3.883, *p* ​< ​0.01; 24 ​h–0.625 ​mg/kg: *t*(8) ​= ​3.3, *p* ​< ​0.05; 1.25 ​mg/kg: *t*(7) ​= ​3.351, *p* ​< ​0.05; 2.5 ​mg/kg: *t*(7) ​= ​4.692, *p* ​< ​0.01 ​**g**: Experimental scheme: BD 1047, HBK-15, and MK-801 were administered *ip* before the familiarization phase to assess encoding, and before the test phase to assess retrieval (created with BioRender.com). **h,i**: The effect of HBK-15 alone and in combination with BD 1047 on novel object exploration time during the encoding (**h**) and retrieval (**i**) phase, evaluated 24 ​h after the familiarization phase. The data are presented as means ​± ​SD. Statistical analysis: one-sample *t*-test, ∗*p* ​< ​0.05, ∗∗*p* ​< ​0.01, ∗∗∗*p* ​< ​0.001 *vs* chance level ​= ​10s; BD 1047+HBK-15+MK-801 group - acquisition: t(9) ​= ​0.1938, *p* ​= ​0.8506; retrieval: t(9) ​= ​0.1788, *p* ​= ​0.862 n ​= ​8–10 mice per group.

HBK-15 was administered *ip* 30 ​min before the familiarization session or the test phase to evaluate whether the tested compound affected the selected learning phases (Fig. 2a). MK-801 was injected *ip* 15 ​min after the administration of the tested compound. Moreover, we investigated how HBK-15 affected the animals' performance in various time intervals – the gap between acquisition and test phase was 15 ​min, 4 ​h, and 24 ​h (encoding process) or 4 ​h and 24 ​h (retrieval process).

To investigate whether the anti-amnesic properties of HBK-15 depend on sigma-1 receptors, the chaperone proteins were blocked using the reference antagonist BD 1047, administered *ip* either 45 ​min before the familiarization session, or 45 ​min prior to the test phase (Fig. 2g). HBK-15 was given 15 ​min after the sigma-1 receptor antagonist, followed by an *ip* injection of MK-801 15 ​min later. No reference compounds were included in this test, as MK-801 reliably induces recognition-memory deficits in this task, and the aim was to separate acquisition *vs* retrieval.

#### Morris water maze

**Fig. 3:**
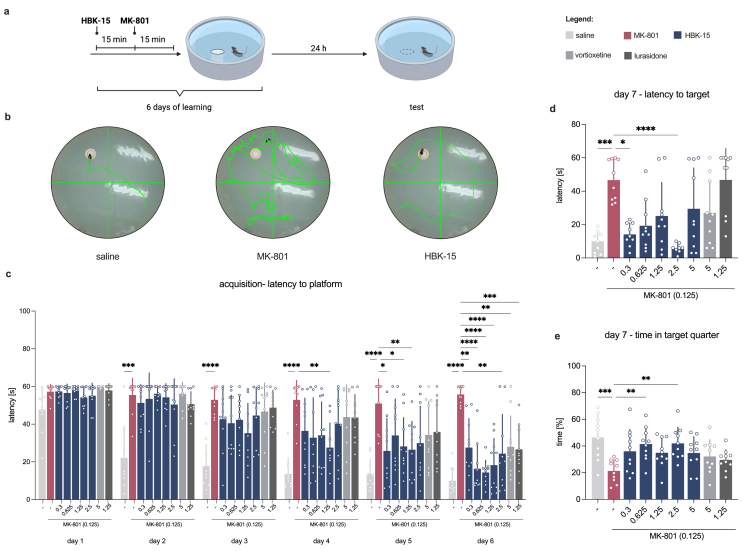
**HBK-15 reversed MK-801-induced spatial learning and memory deficits in the Morris water maze. a:** Experimental scheme: HBK-15 and MK-801 were administered intraperitoneally *(ip)* 30 ​min before the first trial for six consecutive days, and on the seventh day of the experiment, no treatment was administered (created with BioRender.com). **b**: Representative path tracing of the Morris water maze for saline, MK-801, and HBK-15-treated groups during the last day of training. **c:** The effect of HBK-15 on the latency to find a platform in the acquisition phase. The data are presented as means of four trials ​± ​SD. Statistical analysis: two-way ANOVA for repeated measurements with Greenhouse-Geisser correction (Bonferroni *post hoc*), ∗*p* ​< ​0.05, ∗∗*p* ​< ​0.01, ∗∗∗*p* ​< ​0.001, ∗∗∗∗*p* ​< ​0.0001; latency - time: *F*(3.659,296.3) ​= ​125.2, *p* ​< ​0.0001; treatment: *F*(8,81) ​= ​9.322, *p* ​< ​0.0001; interaction: *F*(40,405) ​= ​3.286, *p* ​< ​0.0001; n ​= ​10 mice per group. **d,e**: The influence of HBK-15 on the latency to the first crossing of the target zone (**d**) and the percentage of time spent in the target quarter (**e**) in the probe test (seventh day of the experiment). The data are presented as means ​± ​SD. Statistical analysis: one-way ANOVA (Bonferroni *post hoc*; **e**), Welch ANOVA (Dunnett's T3 *post hoc*; **d**), ∗*p* ​< ​0.05, ∗∗*p* ​< ​0.01, ∗∗∗*p* ​< ​0.001, ∗∗∗∗*p* ​< ​0.0001, latency – *W*(8,31.34) ​= ​18.63, *p* ​< ​0.0001; percentage of time – *F*(8,81) ​= ​3.565, *p* ​= ​0.0014; n ​= ​8–10 mice per group.

*Acquisition phase (days 1-6):* Animals were placed in a circular pool (Panlab-Harvard Apparatus, Spain) filled with opaque water at a temperature of 24 ​± ​1 ​°C and were learned to find a submerged platform using the visual cues around the tank. Each day, the mouse needed to complete four trials separated by 15-min time intervals. If the animal did not find the platform within 60 ​s, it was gently guided to it and left for 15 ​s. HBK-15 was administered *ip* 30 ​min before the first trial for six consecutive days, and MK-801 was injected *ip* 15 ​min later (Fig. 3a–S5a). Vortioxetine (5 ​mg/kg) and lurasidone (1.25 ​mg/kg) were administered as reference compounds, based on prior reports of anti-amnesic activity in rodents and patients [50,51]. The following parameters were collected: the latency to enter the platform (i.e., escape latency), the total distance, and the swimming speed.

*Probe test*: 24 ​h after the last training session, no compounds were administered, and the platform was removed from the pool. Each mouse swam during a 60-s trial, and the following parameters were collected and analyzed: the latency and the covered distance to the target zone (where the platform was previously located), the percentage of time spent in the target quarter, and the swimming speed.

### Neurotransmitter levels

#### Tissue homogenates

*Tissue and homogenates preparation:* 30 ​min after acute administration of MK-801, HBK-15, their combination, or saline, naïve mice were sacrificed, and their brains were rapidly removed and chilled in an ice-cold saline solution. The hippocampi were dissected, frozen, and stored at −80 ​°C until the assay. On the day of experiments, tissues were thawed on ice and homogenized (1:10 w/v) in buffer containing 50 ​mM Tris-HCl, 150 ​mM NaCl, 2 ​mM EDTA, and 0.32 ​M sucrose using a bead homogenizer (Bead Rupture elite, Omni International, USA).

*Analytical method:* Concentrations of dopamine (DA), serotonin (5-HT), acetylcholine (ACh), glutamate (Glu), histamine (HIS), and norepinephrine (NE) in the hippocampi were measured by the liquid chromatography-tandem mass spectrometry method (LC-MS/MS). After deproteinization and centrifugation of homogenate samples, supernatants were transferred into the autosampler vials. Analytes were separated on the XBridge HILIC (2.1 ​× ​150 ​mm, 3.5 ​μm, Waters, USA) analytical column using an Exion LC AC HPLC system (Sciex, USA). The mobile phase consisted of 0.1 ​% formic acid in water and 0.1 ​% formic acid in acetonitrile mixed at the ratio of 30/70 (v/v). A QTRAP 4500 (Sciex, USA) tandem mass spectrometer equipped with an electrospray ionization (ESI) was operated at unit resolution, monitoring the transitions presented in Table S2. Corresponding deuterated internal standards were used for analyte quantification. Calibration curves were linear in the range from 1 to 1000 ​ng/ml for ACh, 5-HT, and DA; from 2.5 to 1000 ​ng/ml for HIS; from 10 to 1000 ​ng/ml for Glu, and from 25 to 1000 ​ng/ml for NE. Then, concentrations of neurotransmitters in the analyzed samples were calculated per g of brain tissue by applying appropriate dilution factors.

#### Microdialysates

*Surgery:* Animals were anesthetized with 2.5 ​% isoflurane (5 ​% for induction) and guide cannulas (MAB 10.8.IC, AgnTho's AB, Sweden) were implanted over the hippocampus (AP – 1.93 ​mm; ML ​+ ​1.5 ​mm; DV – 1.8 ​mm from the bregma; Fig. S2a), according to the stereotaxic atlas of Franklin and Paxinos [52]. After surgery, mice were housed in a high-roofed cage with *ad libitum* access to water and food and monitored for recovery (normal eating, drinking, and defecation).

*Microdialysis:* Following a 3-day recovery period, microdialysis probes (MAB 10.8.1.PES with cut off: 6 kD, AgnTho's AB, Sweden) were connected to the syringe pump (Univentor 864 Syringe Pump, AgnTho's AB, Sweden) that delivered aCSF composed of (mM) 147 NaCl, 4 KCl, 2.2 CaCl_2,_ and 1.0 MgCl_2_ at a flow rate of 1 ​μl/min. The monitoring of extracellular levels of neurotransmitters has been performed in freely moving animals. After a 2-h stabilization period, a baseline sample was collected for 30 ​min. Thereafter, HBK-15 at the dose of 2.5 ​mg/kg was injected *ip,* and three samples were further collected every 30 ​min. Placement of dialysis probes was verified post-mortem in coronal sections (Fig. S2b).

*Analytical method:* Concentrations of ACh and Glu in microdialysis samples from the mouse hippocampus were measured by the LC-MS/MS method similar to the one described above, with some minor modifications. The sample preparation procedure involved the addition of 3 ​μl of an internal standard (IS) mixture to 30 ​μl of dialysate. Then, samples were vortex mixed, transferred to autosampler vials, and injected into the LC-MS/MS system. Analytes were separated using gradient elution with the mobile phase consisting of 25 ​mM ammonium formate (pH ​= ​3.5) in water and acetonitrile. Calibration curves were prepared in the aCSF and were linear in the range from 1 to 1000 ​ng/ml for ACh and Glu.

### Electrophysiology *ex vivo*

*Hippocampal slices:* The hippocampus was dissected in ice-cold aCSF, and slices (400 ​μm thick) were cut with a McIlwain tissue chopper and allowed to recover functionally and energetically for 1 ​h in a resting chamber as previously described [53].

*Field postsynaptic potentials (fEPSPs) recording*: fEPSPs were recorded through an extracellular microelectrode placed in the *stratum radiatum* of the CA1 area (Fig. S3a–b). Stimulation was delivered through an electrode placed on the Schaffer collateral–commissural fibers, in the *stratum radiatum* near CA3–CA1 border (Fig. S3a) as previously described [53].

*Long-term potentiation (LTP) induction and quantification:* Stimulation was delivered alternatively to two independent pathways through bipolar concentric electrodes placed on Shaffer collateral/commissural fibers in *stratum radiatum* (Fig. S3a). A θ-burst protocol was induced, consisting of four trains of 100 ​Hz, and four stimuli, separated by 200 ​ms as previously described (Fig. S3c) [53]. To evaluate the effects of the tested compounds upon LTP, each individual drug was added to the superfusion bath before induction of LTP and remained in the bath up to the end of the experiment. In the experiments where both compounds were tested, HBK-15 was added 15 ​min before MK-801 and remained until the end of the experiments.

### Electrophysiology *in vivo*

*Surgery:* Animals (10 male BALB/c mice) were anesthetized with 1–1.5 ​% isoflurane (3–5 ​% for induction) and placed in a stereotaxic frame (SR-5M-HT, Narishige), and body temperature was maintained at 37 ​°C (50-7221-F, Harvard Bioscience). The scalp was shaved, cleaned (70 ​% ethanol, iodopovidone), and analgesia was provided (Naropin 0.2 ​%, 0.08 ​ml *sc* at the incision site; Rimadyl 0.01 ​%, 0.05 ​ml *sc* in the back). After exposing the skull, multiple bone screws were implanted, with one over the cerebellum serving as ground/reference. A craniotomy was made (AP −2 ​mm, ML +1.5 ​mm from bregma), and a bipolar wire electrode (130.3 ​± ​11.8 ​kΩ ​at 1 ​kHz, AISI 302, 0.1 ​mm diameter, GoodFellow) was inserted into the hippocampal CA1 (−1.5 ​mm from the cortical surface). The site was sealed (Kwik-Sil, World Precision Instruments), and the electrode was secured with dental cement. Mice were housed in pairs with *ad libitum* access to water and food.

*In vivo pharmacology & electrophysiology:* Recordings took place in a plexiglass box (30 ​× ​30 ​× ​40 ​cm). Signals were monitored at 1 ​kHz (RHX, Intan Technologies) via a headstage (C3334, RHD 16-channel, Intan) connected to an interface board (C3100, RHD USB Interface). After habituation, 2 ​h of electrophysiological signals were recorded under four treatments: (1) saline ​+ ​saline, (2) saline ​+ ​MK-801 (0.125 ​mg/kg), (3) HBK-15 (2.5 ​mg/kg) ​+ ​saline, (4) HBK-15 ​+ ​MK-801. Following a 15 ​min baseline, the first intraperitoneal injection was given, and 15 ​min later the second injection was administered contralaterally (Fig. 5a). Recordings continued for 1.5 ​h after the second injection. Each mouse received all four treatments in random order, with at least one day between sessions.

*Histology:* After experiments, mice were deeply anesthetized (pentobarbital/lidocaine) and perfused transcardially with PBS followed by 4 ​% paraformaldehyde in 0.1 ​M phosphate buffer (pH 7.4). Brains were postfixed overnight at 4 ​°C, then placed in 30 ​% sucrose for at least two days. Coronal sections (100 ​μm) were stained with DAPI (1:1000, Sigma-Aldrich) and mounted (Fluoromount-G, ThermoFisher). Images were captured under an epifluorescence microscope (Eclipse E600, Nikon; Fig. S4).

*Data analysis:* Offline analyses were performed in MATLAB R2022a. Hippocampal LFPs were used to compute theta (4–10 ​Hz) and gamma (30–45 ​Hz) power via spectrograms (10 ​s window, 5 ​s overlap). Power values were normalized by total power ≤40 ​Hz, and Z-scores were calculated using baseline signals (5–15 ​min). Treatment effects were assessed by averaging Z-scores from 30 to 60 ​min after the second injection. Phase-amplitude coupling (PAC) between theta and gamma was estimated by bandpass filtering (4–10 ​Hz, 30–45 ​Hz) and applying the Hilbert transform. The modulation index was calculated as the ratio of maximum to minimum gamma amplitude across theta phases, then Z-scored based on baseline. Treatment effects on PAC were evaluated from 30 to 60 ​min after the second injection.

### Statistical analysis

Results are presented as mean ​± ​standard deviation (SD) or standard error of mean (SEM) for parametric analysis, or median and interquartile range (IQR) for non-parametric analysis. Data normality and homogeneity were verified via the Shapiro-Wilk and Brown-Forsythe tests. Various ANOVAs, with appropriate *post hoc* tests (Dunnett's, Tukey's, Bonferroni), were used for group comparisons. Non-parametric Kruskal-Wallis analysis with Dunn's *post hoc* test and Welch ANOVA test with Dunnett's T3 test were used when assumptions were not met. A one-sample *t*-test compared novel object exploration time to chance level in an object recognition test. A *p-*value <0.05 signified significance. Analyses were done with GraphPad 9.5.0 and MATLAB (R2022a).

## Results

### HBK-15 displays high affinity and functional activity at the sigma-1 receptor

We first profiled HBK-15 across sigma receptors, glutamatergic targets, ion channels, and the 5-HT_3_ receptor, covering a panel of systems implicated in memory regulation. Radioligand binding revealed a marked preference for the sigma-1 receptor (*p*Ki ​= ​7.604, Ki ​= ​24.86 ​nM), with weaker affinity for sigma-2 receptor (*p*Ki ​= ​6.695, Ki ​= ​201.9 ​nM), and negligible activity at other tested sites (Table 1). To determine whether this translated into functional engagement, we employed a BiP–sigma-1 dissociation assay (Fig. 1a). HBK-15 induced a concentration-dependent release of BiP from sigma-1 receptors (IC_50_ of 1016 ​nM; Fig. 1b), consistent with intrinsic agonist activity. Together, these findings show that HBK-15 binds with high affinity to the sigma-1 receptor and exhibits intrinsic agonist activity in a BiP dissociation assay.

**Table 1:** *In vitro* binding assays for HBK-15.

Molecular target	% inhibition of control-specific binding (10^−6^ ​M)
**sigma 1**	**82.1**
sigma 2	65.5
KA	−8
AMPA	−9
NMDA (Glu)	7
NMDA (Gly)	−4
NMDA (MK-801)	−6
NMDA (PA)	1
mGluR1	−6
mGluR2	−22
mGluR5	−9
Cav1.2 (phenylalkylamine)	57
Cav2.2 (*ω*-conotoxin)	2
Cav2.2 (gabapentin)	−6
5-HT_3_	−1

HBK-15 was tested at concentrations 10^−6^ ​M. The results are presented as % inhibition of control-specific binding in the presence of HBK-15. Results showing an activity >60 ​% were considered to represent significant effects of the test compound; results showing an inhibition between 25 ​% and 60 ​% indicate moderate to weak effect; results showing an inhibition <25 ​% are not considered significant and mostly attributable to the variability of the signal around the control level. Binding studies were performed commercially in Eurofins Laboratories (Poitiers, France).

The binding sites forN-methyl-D-aspartate (NMDA) receptors are presented in brackets. Glu – glutamate, Gly – glycine, PA – polyamines, KA - kainate, AMPA - α-amino-3-hydroxy-5-methyl-4-isoxazolepropionic acid

### HBK-15 prevents MK-801-induced recognition memory deficits in both acquisition and retrieval

We next evaluated the effects of HBK-15 on recognition memory using the object recognition test under NMDA receptor blockade with MK-801. HBK-15 prevented acquisition deficits, improving object recognition at 15 ​min, 4 ​h, and 24 ​h across the three tested doses (Fig. 2b–d). During retrieval, HBK-15 attenuated deficits, increasing novel object exploration at multiple doses after both 4 ​h and 24 ​h (Fig. 2e-f). Together, these results demonstrate that HBK-15 alleviated MK-801-induced recognition memory impairments across the acquisition and retrieval phases.

### The anti-amnesic effects of HBK-15 require sigma-1 receptor activation

To determine whether the cognitive effects of HBK-15 require sigma-1 receptor activation, we administered the selective sigma-1 receptor antagonist BD 1047 together with HBK-15. We used HBK-15 at 2.5 ​mg/kg, the higher dose that consistently showed anti-amnesic efficacy across behavioral paradigms and learning phases, and had also proven effective in our previous studies [18,21], while 1 ​mg/kg BD 1047 was selected based on previously reported effectiveness [30]. BD 1047 abolished the anti-amnesic effects of HBK-15 across acquisition and retrieval phases, reducing novel object exploration to chance levels (10 ​s; Fig. 2h-i). Together, these data confirm that the anti-amnesic effects of HBK-15 require sigma-1 receptor activation.

### HBK-15 reverses MK-801-induced deficits in spatial learning and memory

To expand our understanding of HBK-15's anti-amnesic properties, we evaluated another memory domain, spatial memory, in the Morris water maze. MK-801 disrupted spatial learning (acquisition), increasing latency and distance to locate the hidden platform (Fig. 3b–c, S5b). HBK-15 attenuated these deficits, with improvements in latency emerging by day 4 and all doses enhancing performance by day 6 (Fig. 3c). By contrast, vortioxetine and lurasidone improved performance only on day 6 (Fig. 3c). For path length, significant effects of HBK-15 were observed on day 6 (Fig. S5b). Considering swimming speed, MK-801 reduced it, whereas HBK-15 increased it, at two doses starting from day 5, and at all tested doses by day 6 (Fig. S5c).

In the probe test performed 24 ​h later without treatment, HBK-15 reversed MK-801-induced impairments in spatial memory. Treated animals showed shorter latency and distance to the target zone and increased time in the target quadrant (Fig. 3d–e, S5d). No differences in swimming speed were observed between groups during the probe (Fig. S5e). During the probe test, neither vortioxetine nor lurasidone reversed the MK-801-induced impairments in any parameter. Thus, HBK-15 prevented disturbances in both spatial learning and retrieval, whereas vortioxetine and lurasidone showed only partial efficacy on a single learning day.

### HBK-15 modulates hippocampal glutamatergic and cholinergic signaling under NMDA receptor blockade

We first measured hippocampal neurotransmitter levels in naïve animals. A single administration of HBK-15 (0.3–5 ​mg/kg) produced no significant changes in serotonin, noradrenaline, histamine, dopamine, glutamate, or acetylcholine in hippocampal homogenates (Table S3) or in glutamate and acetylcholine levels measured by microdialysis (Table S4).

**Fig. 4:**
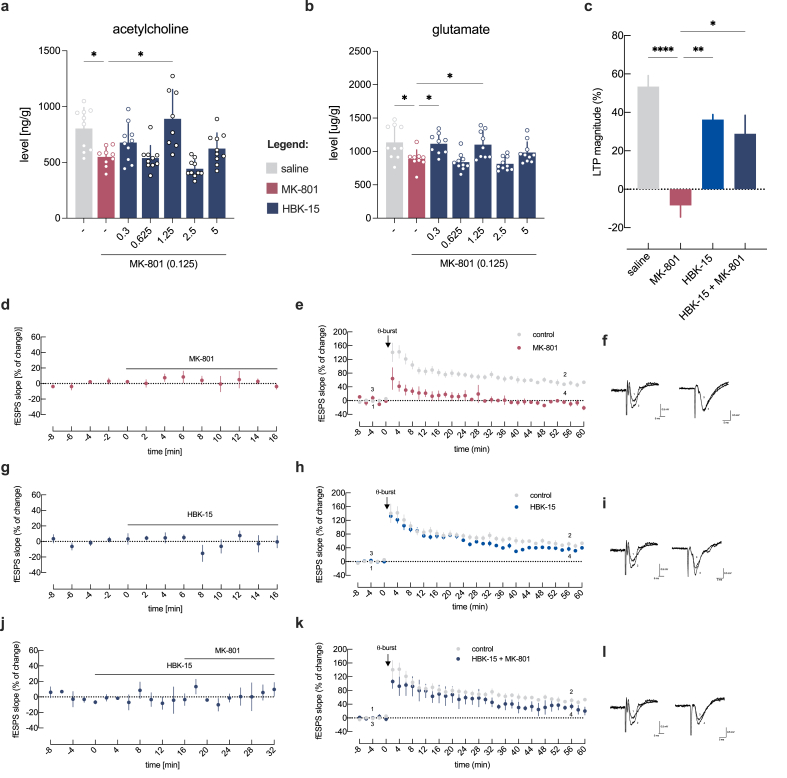
**HBK-15 reverses MK-801–induced impairments in hippocampal plasticity and neurotransmitter signaling. a,b:** The effect of HBK-15 on the level of acetylcholine (**a**) and glutamate (**b**) in the hippocampus of MK-801-treated mice. Data are presented as means ​± ​SD. Statistical analysis: one-way ANOVA (Dunnett's *post hoc;***b**) or Welch ANOVA (Dunnett's T3 *post hoc*; **a**), ∗*p* ​< ​0.05; acetylcholine: *W*(6,31.81) ​= ​8.708, *p* ​< ​0.0001, glutamate: *F*(6,60) ​= ​6.452, *p* ​< ​0.0001, n ​= ​9–10 mice per group **c:** The magnitude of LTP (change in the fEPSP slope at 50-60 ​min) induced by θ-burst stimulation in relation to pre-θ-burst values (0 ​%) in the absence of any drug or in the presence of MK-801, HBK-15, or both drugs together. **d,g,j:** The averaged time courses of changes in fEPSP slope induced by the application of 10 ​μM of MK-801 (**d**; n ​= ​4), or 1 ​μg/ml of HBK-15 (**g**; n ​= ​5) or HBK-15 (1 ​μg/ml) followed by the addition of MK-801 (10 ​μM) (**j**; n ​= ​3) **e,h,k:** The averaged time course of changes in fEPSP slope observed after a theta (θ) burst stimulation in control conditions (first pathways) without any drug (grey circles) or (second pathway) in the presence of 10 ​μM of MK-801 (**e**, red circles) or 1 ​μg/ml of HBK-15 (**h**, blue circles) or both drugs together (**k**, navy circles). **f,i,l:** Traces obtained in representative experiments in **e,h,k,** respectively. Each trace is the average of six consecutive responses obtained immediately before (1) and after (2) θ-burst stimulation in the control condition or before (3) and after (4) θ-burst in the presence of MK-801 (**f**), HBK-15 (**i**), or both drugs together (**l**). Each trace is composed of the stimulus artifact, followed by the presynaptic volley and the fEPSP; recordings obtained in the same experiment are superimposed. All data are presented as means ​± ​SEM. Statistical analysis: one-way ANOVA (Tukey's *post hoc*), ∗*p* ​< ​0.05, ∗∗*p* ​< ​0.01, ∗∗∗∗*p* ​< ​0.001; (*F*(3,15) ​= ​15.24, *p* ​< ​0.001, control: 53.38 ​± ​5.77, n ​= ​8; MK-801: -8.40 ​± ​6.08, n ​= ​3; HBK-15: 36.27 ​± ​2.63, n ​= ​5; HBK-15+MK-801: 28.88 ​± ​9.61, n ​= ​3).

In contrast, under MK-801 treatment, HBK-15 altered neurotransmitter levels in a dose-specific manner. At 0.3 ​mg/kg, it increased glutamate, whereas at 1.25 ​mg/kg, it elevated both glutamate and acetylcholine (Fig. 4a-b). Levels of other neurotransmitters remained unchanged under all conditions (Table S5). Together, these results indicate that HBK-15 does not affect basal neurotransmission but enhances glutamatergic and cholinergic signaling under conditions of NMDA receptor hypofunction.

### HBK-15 rescues hippocampal LTP from MK-801–induced impairments

To examine whether HBK-15 influences synaptic plasticity disrupted by MK-801, we conducted *ex vivo* electrophysiological recordings in hippocampal slices. As shown in Fig. 4d and g, neither MK-801 (10 ​μM) nor HBK-15 (1 ​μg/mL) alone substantially affected baseline fEPSP slopes. The combined application of both compounds under baseline conditions also resulted in no observable changes (Fig. 4j).

Application of MK-801 significantly reduced LTP magnitude compared to control slices (Fig. 4c,e-f), consistent with impairments observed in behavioral memory tasks. Remarkably, HBK-15 restored LTP magnitude toward control levels in the presence of MK-801 (Fig. 4c,k-l) and significantly improved potentiation relative to MK-801 alone. HBK-15 on its own did not alter LTP magnitude substantially compared with control (Fig. 4c,h-i). Together, these findings show that HBK-15 counteracted MK-801–induced impairments in hippocampal LTP, without affecting plasticity under control conditions.

### HBK-15 enhances hippocampal theta-gamma coupling *in vivo*

**Fig. 5:**
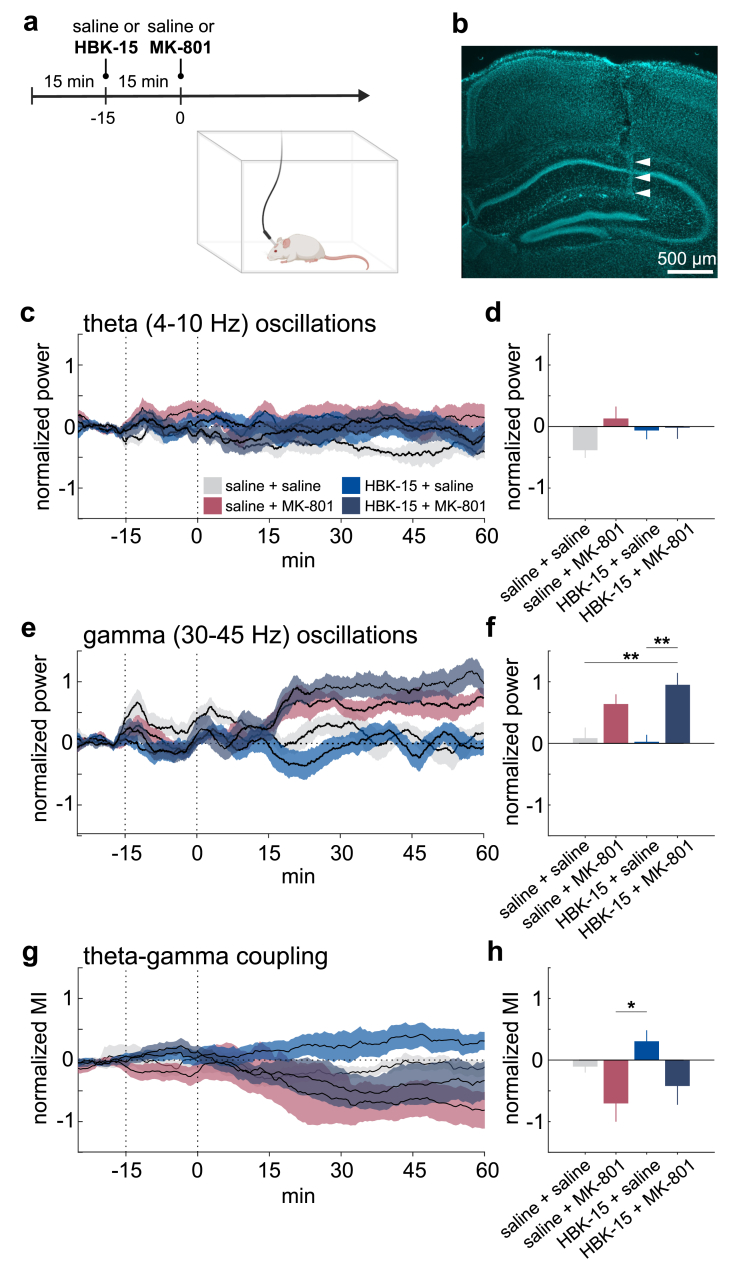
**HBK-15 modulated hippocampal oscillations *in vivo*. a:** Experimental design and schematic drawing of *in vivo* electrophysiological recording. **b:** Histological confirmation of the electrode position. **c:** Normalized theta (4–10 ​Hz) power across all conditions: saline ​+ ​saline, saline ​+ ​MK-801 (0.125 ​mg/kg), HBK-15 (2.5 ​mg/kg) ​+ ​saline, and HBK-15 ​+ ​MK-801. **d:** The average of the normalized theta power between 30 and 60 ​min after the second injection, one-way ANOVA with *post hoc* Tukey's HSD test, *F*(3,36) ​= ​1.75, *p* ​= ​0.17, n ​= ​10. **e:** Normalized gamma (30–45 ​Hz) power across all conditions. **f:** The average of the normalized gamma power between 30 and 60 ​min after the second injection. ∗∗*p* ​< ​0.01, one-way ANOVA with *post hoc* Tukey's HSD test, *F*(3,36) ​= ​7.59, *p* ​< ​0.01, n ​= ​10. **g:** Normalized modulation index (MI) of the phase-amplitude coupling between theta and gamma oscillations across conditions. **h:** The average of the normalized MI between 30 and 60 ​min after the second injection. ∗*p* ​< ​0.05, one-way ANOVA with *post hoc* Tukey's HSD test, *F*(3,36) ​= ​3.40, *p* ​< ​0.05, n ​= ​10. All data are presented as means ​± ​SEM.

To examine the effects of HBK-15 and MK-801 on hippocampal activity *in vivo*, we conducted *in vivo* electrophysiological recordings from the dorsal hippocampal CA1 region in a freely behaving condition under four treatment options: 1) saline ​+ ​saline, 2) saline ​+ ​MK-801, 3) HBK-15 ​+ ​saline and 4) HBK-15 ​+ ​MK-801 (Fig. 5a). The electrode position was confirmed in the CA1 region based on *post* *mortem* histological analysis (Fig. 5b, S4).

Because theta and gamma oscillations are prominent rhythms in the hippocampus and their interaction has been linked to memory processing [[54], [55], [56]], we first analyzed the power of theta (4–10 ​Hz) and gamma (30–45 ​Hz) oscillations from hippocampal local field potentials (LFPs) (Fig. 5c–f). Thirty to 60 ​min after the second injection, we did not observe any significant effect of treatment on theta power (Fig. 5c and d). On the other hand, we observed a significant increase in gamma power in the administration of both HBK-15 and MK-801 (Fig. 5e-f), while MK-801 administration alone tended to increase gamma power too.

To further explore the effect of HBK-15 on hippocampal oscillations, we computed the phase-amplitude coupling (PAC) between theta and gamma oscillations (Fig. 5g–h), a phenomenon implicated in hippocampal memory function [[57], [58], [59]]. Intriguingly, we observed opposing trends in the PAC between HBK-15 and MK-801 administrations (Fig. 5g): HBK-15 tended to facilitate the PAC, whereas MK-801 tended to disorganize the coupling. We confirmed this by comparing the normalized modulation index (MI) between HBK-15- and MK-801-treated conditions (Fig. 5h). When both drugs were administered, the negative impact of MK-801 tended to be improved. Overall, HBK-15 can modulate the coordination of hippocampal oscillations *in vivo*.

## Discussion

We found that HBK-15 reversed memory impairments induced by NMDA receptor hypofunction, a mechanism strongly implicated in the cognitive symptoms of depression and schizophrenia. The compound restored both recognition and spatial memory across acquisition and retrieval phases. These effects critically depended on sigma-1 receptor activation and were accompanied by restoration of hippocampal LTP and partial normalization of hippocampal theta-gamma coupling. Together, these findings suggest that HBK-15 stabilizes plasticity at both synaptic and network levels, positioning it as a promising candidate for treating memory disturbances in neuropsychiatric disorders.

Despite their profound impact on functional outcome, cognitive deficits in depression and schizophrenia remain largely unaddressed by current pharmacotherapies [60]. Existing treatments primarily target mood or psychotic symptoms, leaving cognitive impairments unresolved and contributing to relapses, poor functional recovery, and chronic disability [1]. This therapeutic gap underscores the need for agents that can directly modulate memory processes. In our earlier work, we identified HBK-15, a methoxyphenylpiperazine derivative with multimodal activity, showing the highest affinity for the 5-HT_1A_ receptor (*p*Ki ​= ​9.0) and lower affinity for 5-HT_7_ (*p*Ki ​= ​7.47), D_2_ (*p*Ki ​= ​7.27), and α_1_ receptors [[21], [22], [23]]. The compound also displayed functional selectivity at 5-HT_1A_ and 5-HT_7_ receptors and produced rapid antidepressant- and anxiolytic-like effects along with preliminary evidence of anti-amnesic efficacy in rodent models [18,20,21,24]. Building on these findings, the present study suggests that sigma-1 receptor activation specifically contributes to HBK-15's anti-amnesic effects. Together, these mechanisms indicate that HBK-15 may extend beyond affective regulation to target memory dysfunction through pathways distinct from conventional antidepressants.

Building on this rationale and given that encoding and retrieval deficits can manifest independently in depression and schizophrenia, we designed our experiments to probe both phases. Clinical studies show that patients with schizophrenia exhibit pronounced impairments in relational encoding and recollection, whereas in major depression, retrieval failures often coexist with encoding abnormalities [28,29]. This separation is also reflected in clinical trial standards, where verbal learning tests typically distinguish acquisition from delayed recall or recognition [61,62]. To align with these constructs, we modeled encoding with object recognition acquisition and Morris water maze training, and retrieval with object recognition retrieval and the water maze probe test. This translational mapping enabled us to determine whether HBK-15 benefits specific phases of memory or exerts broader efficacy across both. Additionally, we varied the retention interval to dissociate HBK-15's effects across distinct phases of memory formation and recall. The 15-min condition primarily indexes encoding and early consolidation, whereas the 24-h condition reflects retrieval of a stabilized engram. The inclusion of a 4-h interval provided an intermediate readout of early long-term memory, bridging these endpoints. This design mirrors clinical assessments, in which immediate learning, short-delay recall, and delayed recall/recognition are evaluated separately (e.g., HVLT-R, RAVLT). Accordingly, HBK-15's efficacy across both short- and long-delay intervals indicates benefits spanning from encoding/early consolidation to retrieval, rather than being restricted to a single phase.

To benchmark HBK-15 against established treatments, we included vortioxetine and lurasidone in the Morris water maze test, as both have shown cognitive-enhancing effects in humans (reviewed in Refs. [63,64]). Vortioxetine is a multimodal antidepressant acting as a 5-HT_1A_ receptor agonist and 5-HT_3_/5-HT_7_ receptor antagonist with serotonin transporter inhibition [65], whereas lurasidone is an atypical antipsychotic combining D_2_ and 5-HT_2A_ receptor antagonism with 5-HT_7_ blockade [66] - mechanisms associated with anti-amnesic activity. Both compounds produced only a transient improvement during acquisition that did not extend to retrieval. Neither compound fully reversed MK-801-induced spatial deficits, in contrast to the consistent efficacy observed with HBK-15. This mirrors clinical findings, where vortioxetine and lurasidone provide at best mixed or modest cognitive benefits [63,67], underscoring HBK-15's broader and more consistent efficacy.

To address potential mechanisms, we investigated whether the anti-amnesic activity of HBK-15 involves sigma-1 receptor, a mechanism tightly linked to glutamatergic plasticity. The compound showed high affinity for the sigma-1 receptor and acted as a moderate agonist, displaying lower efficacy than the reference agonist PRE-084 (44 ​nM). As the sigma-1 receptor is a ligand-regulated chaperone at mitochondria-associated endoplasmic reticulum membranes (MAMs) that stabilizes IP_3_R-dependent ER-to-mitochondria Ca^2+^ transfer and supports glutamatergic plasticity [43], we hypothesized that its engagement would alleviate memory deficits linked to NMDA receptor dysfunction in depression and schizophrenia [68]. Our behavioral data confirmed sigma-1 receptor-dependent rescue. Consistent with this framework, selective sigma-1 receptor agonists such as PRE-084 and SA4503 reliably counteract MK-801-induced amnesia and enhance hippocampus-dependent memory [[69], [70], [71]]. However, not all neuropsychiatric drugs with sigma-1 affinity demonstrate strong or consistent cognitive benefits across different paradigms. PET studies have shown that fluvoxamine (a selective serotonin reuptake inhibitor (SSRI) antidepressant) activates sigma-1 receptors in the human brain, and clinical findings suggest that it can improve cognitive domains, such as verbal memory and attention, in patients with remitted depression [[72], [73], [74]]. However, at therapeutic doses also produces ∼80 ​% serotonin-transporter occupancy, rapidly elevating extracellular serotonin - an effect that may shift anxiety/motivation and prefrontal-hippocampal gating during encoding/retrieval, potentially masking subtler sigma-1-driven plasticity signals [75,76]. Thus, while clinical data support the potential of fluvoxamine to exert cognitive-enhancing effects in selected patient populations [[72], [73], [74]] its profile may lead to varied outcomes depending on context and task demands. In contrast, opipramol (a tricyclic antidepressant used clinically for anxiety) likewise activates sigma-1 receptors, but off-target H_1_ antagonism leads to sedation and attentional costs that may offset mnemonic benefits [77]. Haloperidol, in turn, shows high sigma-1 affinity but antagonistic action, consistent with its cognitive impairing profile in schizophrenia [78,79]. Finally, donepezil, an acetylcholinesterase inhibitor widely used in Alzheimer's disease, also engages sigma-1 receptors, but has failed to improve cognition in depression or schizophrenia, underscoring that sigma-1 activity alone is insufficient to reverse memory disturbances in these disorders [80,81]. In contrast, HBK-15 provides moderate sigma-1 receptor agonism without serotonin-transporter inhibition [18] or histamine receptor antagonism [82], avoiding liabilities that can interfere with memory performance. Instead, HBK-15 combines sigma-1 engagement with a multimodal receptor profile [[18], [19], [20], [21], [22]], binding with high affinity to the 5-HT_1A_ receptor and, to a lesser extent, to the 5-HT_7_ receptor. Engagement of these targets antagonizes cAMP signaling, a mechanism associated with procognitive effects [18,21,83]. This pharmacological constellation may therefore be better suited to support memory encoding after NMDA receptor disruption. At the mechanistic level, sigma-1 receptor-mediated control of Ca^2+^ flux at mitochondria-associated ER membranes could sustain NMDA receptor-dependent plasticity. Together, these properties may help explain why HBK-15 showed anti-amnesic activity in our paradigm, whereas not all drugs targeting the sigma-1 receptor consistently improve memory. Beyond its ability to alleviate cognitive deficits, sigma-1 receptor engagement may also translate into broader therapeutic benefits. Sigma-1 receptor agonism has been linked to antidepressant- and antipsychotic-like effects in both preclinical and clinical contexts [84,85]. In line with this, our previous studies demonstrated that HBK-15 produced rapid antidepressant-like effects in animal models [20,21,24], suggesting that its sigma-1 receptor activity may contribute not only to cognitive improvement but also to the treatment of depressive symptoms. However, as HBK-15 did not exhibit antipsychotic-like properties in rodents [21], its utility in schizophrenia may be limited to addressing cognitive dysfunction rather than core psychotic features.

Interestingly, HBK-15 did not follow a simple linear dose-response across memory paradigms. Instead, it produced binary, biphasic, or inverted U-shaped effects depending on the memory type and phase examined. Such non-linear profiles are well documented in neuropharmacology, particularly among ligands acting on serotonergic, dopaminergic, and cholinergic systems, and are thought to reflect both receptor-level mechanisms and the dynamic regulation of neurotransmitter networks [[86], [87], [88]]. In this context, sigma-1 receptor ligands are also known to exhibit non-linear dose–response relationships arising from complex receptor dynamics, including oligomerization, intracellular translocation, and cross-talk with neurotrophic signaling pathways [84,89]. The alignment of HBK-15's behavioral profile with these established sigma-1 receptor properties reinforces the conclusion that sigma-1 receptor engagement underlies its cognitive-enhancing activity.

Learning and memory critically depend on neurotransmitter regulation, and MK-801-induced NMDA receptor blockade disrupts both glutamatergic and cholinergic signaling [90,91] consistent with our results. In our study, HBK-15 alone did not alter basal hippocampal transmitter levels, but under MK-801, it increased glutamate and acetylcholine at selected doses, restoring them toward control values. Because glutamate and acetylcholine are especially critical for early encoding, these shifts may help explain HBK-15's effects during acquisition. However, transmitter normalization did not map directly onto behavioral outcomes, indicating that transmitter modulation alone is neither necessary nor sufficient for the anti-amnesic effect. Instead, activation of the sigma-1 receptor appears to set the stage for more durable plasticity by regulating calcium flow between the ER and mitochondria and stabilizing NMDA receptor function.

Given this, we next examined whether HBK-15 could influence synaptic mechanisms more directly. LTP, a fundamental substrate of learning and memory [92,93], is reduced in depression [94] and schizophrenia [95] and, as expected, was impaired by acute NMDA receptor blockade with MK-801 in our experiments. HBK-15 reversed this deficit, indicating that its anti-amnesic effects extend to stabilization of synaptic plasticity. Importantly, HBK-15 lacks direct affinity for NMDA and other major glutamate receptors and shows no activity at 5-HT_3_ or nicotinic receptors, with only modest affinity for Cav1.2 channels. This profile suggests that the restoration of LTP occurs indirectly, most likely through activation of the sigma-1 receptor, which regulates ER-mitochondrial calcium transfer to sustain synaptic energy supply and plasticity [17,96]. Through this mechanism, sigma-1 receptor activation can potentiate NMDA receptor responses and facilitate LTP, explaining how HBK-15 rescues synaptic plasticity despite lacking direct glutamatergic actions. Because long-term potentiation is widely regarded as a cellular correlate of encoding, this effect provides a plausible mechanism for HBK-15's ability to improve acquisition.

Our earlier work suggested that HBK-15 might act as a 5-HT_3_ receptor antagonist in a biofunctional assay (isolated guinea pig ileum contraction; 5-HT_3_ receptor-driven response) [20], but this was not supported by the current radioligand binding experiments, which showed no measurable affinity. The discrepancy likely reflects methodological differences: *ex vivo* assays in guinea pig ileum can yield false positives when compounds interfere with voltage-gated sodium or calcium channels, as blocking these channels indirectly suppresses 5-HT_3_ activity [19,97,98]. Thus, the prior signal most likely represented an off-target effect rather than genuine 5-HT_3_ receptor antagonism.

Oscillatory activity provides a complementary level of synaptic plasticity regulation. MK-801 augments gamma oscillations and disrupts hippocampal theta-gamma coupling during spatial working memory tasks [99], mirroring the disturbances reported in patients with schizophrenia during working memory performance [100]. Our findings resonate with these studies, demonstrating that a single administration of MK-801 disrupts this coupling. Interestingly, the co-administration of HBK-15 and MK-801 appeared to improve the theta-gamma coupling, although the differences were not statistically significant. Using only one HBK-15 dose, selected for optimal behavioral performance, may have limited our ability to detect significant improvements. On the other hand, HBK-15's anti-amnesic effects might not solely depend on hippocampal oscillation coupling, and other mechanisms could be at play. Interestingly, when administered independently, HBK-15 promoted the theta-gamma coupling, suggesting the compound's potential to improve learning and memory processes. Several studies associated increased theta-gamma coupling in the hippocampus with enhancements in spatial reference memory [101] or associative memory [102]. As theta-gamma coupling has been linked to retrieval and integration of stored representations [103], HBK-15's ability to promote this coordination raises the possibility that its benefits extend to network-level mechanisms of retrieval as well.

Our study offers valuable insights but has certain limitations. We concentrated on the acute/subchronic MK-801 model, which captures only selected aspects of cognitive impairments in depression and schizophrenia. Still, this model provided a reliable framework to investigate, for the first time, how HBK-15 influences memory deficits and allowed us to explore broader aspects of neuropsychiatric disorders within a single paradigm. We tested the effects of both single and short repeated doses (up to six days), but longer treatment durations are necessary to evaluate the longevity and clinical significance of HBK-15's cognitive benefits. While the present work centered on NMDA receptor hypofunction associated with psychiatric disorders, the anti-amnesic effects of HBK-15 warrant exploration in neurodegenerative settings. Future studies should establish whether its procognitive activity extends to models characterized by NMDA receptor overactivation and excitotoxicity. Addressing these limitations in future studies will enhance the translational relevance of our results.

In conclusion, the multimodal compound HBK-15 emerges as a sigma-1 receptor agonist capable of restoring recognition and spatial memory under NMDA receptor hypofunction. By rescuing hippocampal LTP and stabilizing oscillatory coordination, it engages both synaptic and network-level plasticity to counteract MK-801-induced impairments. These findings not only position HBK-15 as a promising candidate for addressing memory dysfunction in depression and schizophrenia - domains largely neglected by current pharmacotherapies - but also highlight sigma-1 receptor engagement as a tractable mechanism for developing next-generation multi-target directed ligands targeting cognitive symptoms that remain resistant to existing treatments.

## Author contributions

Conceptualization: KS, MJD, SX, SS, KP (lead); Methodology: KS, MJD, SX, SS, TM, KP; Investigation: KS, KL, AJ, MS, SI, MJD, SX, PS, LM, EM, JF, SS, LC, JM, KP; Formal analysis: KS (lead), SI, SS, LC, JM, TM, KP; Data curation: KS, KL, AJ, KP (lead); Resources: MJD, SX, SS, TM, KP; Writing – Original Draft: KS, KL, AJ, MS, SI, MJD, SS, KP (lead); Writing – Review & Editing: KS, KL, AJ, MS (support), SI (support), MJD (support), SX (support), PS (support), LM (support), EM (support), JF (support), SS, LC (support), JM (support), BD, TM, KP (lead); Supervision: MJD, SX, SS, TM, KP (lead); Funding acquisition: MJD, SS, KP (lead).

## Funding

This study was financially supported by the 10.13039/501100004442National Science Centre, Poland (grant number 2019/34/E/NZ7/00454) to KP, Santa Casa da Misericórdia de Lisboa (MB35-2021) to MJD and Medical Research Council (MR/V033964/1) and Horizon2020-ICT (DEEPER, 101016787) to SS. The work has been supported through the European Cooperation in Science and Technology (COST Association), SIGMA1_EUROPE action under No. CA23156.

## Declaration of competing interest

The authors declare no competing interests that could have influenced the work reported in this manuscript.
