# Supplementary material for: Sigma-1-targeting multimodal compound HBK-15 reverses memory deficits and restores hippocampal plasticity under NMDA hypofunction
Source: Neurotherapeutics. 2025 Nov 3;23(1):e00774. doi: 10.1016/j.neurot.2025.e00774 (PMC12976527; doi:10.1016/j.neurot.2025.e00774)
Supplement: Multimedia component 1 [file mmc1.docx]

**Sigma-1-targeting multimodal compound HBK-15 reverses memory deficits and restores hippocampal plasticity under NMDA hypofunction**

Kinga Sałaciak*, Klaudia Lustyk*, Angelika Jagielska*^,^†, Małgorzata Szafarz‡, Sara Inteiro-Oliveira§^,^¶, Maria José Diógenes§^,^¶, Sara Xapelli§^,^¶, Paulina Schnur||, Lucy Morton||, Erin Moran||, Jacques Ferreira||, Shuzo Sakata||, Lucie Crouzier#, Johann Meunier#, Benjamin Delprat#, Tangui Maurice#, Karolina Pytka*

*Laboratory of Experimental Neuropharmacology, Department of Pharmacodynamics, Faculty of Pharmacy, Jagiellonian University Medical College, 9 Medyczna St, 30-688 Krakow, Poland

†Doctoral School of Medical and Health Sciences, Jagiellonian University Medical College, 16 Św. Łazarza St, 31-530 Krakow, Poland

‡Department of Pharmacokinetics and Physical Pharmacy, Faculty of Pharmacy, Jagiellonian University Medical College, 9 Medyczna St, 30-688 Krakow, Poland

§ Centro Cardiovascular da Universidade de Lisboa (CCUL@RISE), Faculdade de Medicina, Universidade de Lisboa, Lisboa, Portugal

¶ GIMM - Gulbenkian Institute for Molecular Medicine, Lisboa, Portugal

||Strathclyde Institute of Pharmacy and Biomedical Sciences, University of Strathclyde, 161 Cathedral St, Glasgow G4 0RE, UK

#MMDN, Univ Montpellier, EPHE, INSERM, Montpellier, France

**Corresponding Author:**

Karolina Pytka

Department of Pharmacodynamics, Faculty of Pharmacy, Jagiellonian University Medical College, 9 Medyczna St, 30-688 Krakow, Poland

Phone number: +48 (12) 620-55-38, e-mail: [karolina.pytka@uj.edu.pl](mailto:karolina.pytka@uj.edu.pl)

**Detailed Materials and Methods**

**Animals**

Depending on the experiment, we used naïve adult male BALB/c (Mossakowski Medical Research Institute, Polish Academy of Sciences or Charles River Laboratories (Barcelona, Spain, or Edinburgh, UK)) or C57BL/6J (Mossakowski Medical Research Institute, Polish Academy of Sciences) weighing 24 ± 2 g (8-10 weeks old). We conducted all studies on male animals to minimize variability related to hormonal fluctuations in females, ensuring consistent and reproducible results. The animals were kept under standard conditions (19–22°C, humidity 55%, 12:12 h light: dark cycle, food, and water *ad libitum*) and used only once in each test. Behavioral experiments were performed between 8 am and 4 pm and evaluated by a trained observer blind to the treatments. Mice were handled for one week before starting the experimental procedures. Animals were randomly allocated to the treatment using a computer-generated sequence, and researchers making measurements on the animals or analyzing the results were blind to the allocation. Moreover, experimental groups were distributed across multiple cages, and the location of the mouse cages in the room was changed each day. Any variation in group sizes resulted from experimental losses, violations of predetermined exclusion criteria, or exclusions based on outlier identification using the ROUT method.

All experiments were performed following the European (2012/707/EU) and Polish, Portuguese, or the United Kingdom Animals (Scientific Procedures) Act of 1986 Home Office regulations (approved by either the Local Ethics Committee for Experiments on Animals in Kraków: 485/2021, 549/2021, 550/2021, 591/2022, 604/2022 or by the Home Office: PPL0688994).

**Experimental procedures**

**S1R/BiP dissociation assay**

The assay was performed following the methodology described by Hayashi & Su [1] with an optimized protocol [2,3]. CHO cells overexpressing a GFP-tagged S1R (GFP-SIR-oe CHO; gift from Dr Tsung-Ping Su and Dr Yuko Yasui, IRP, NIDA/NIH, Baltimore, MD, USA) were maintained in DMEM/Glutamax culture medium supplemented with 10% heat-inactivated FBS. GFP-S1R-oe CHO cells were plated in 12-well plates and treated with the test drug dissolved in culture medium for 30 min at 37°C. The reaction was stopped by removing the culture medium containing the test drug and replacing it with 1 ml of PBS. GFP-S1R-oe CHO cells were harvested and suspended in PBS (pH 7.4), followed by cross-linking with 50 μg/ml of dithiobis succinimidyl propionate (ThermoFisher, France). The reaction was stopped by adding Tris/HCl 50 mM (pH 8.8). After a 15-min incubation on ice, cells were lysed using 50 mM Tris (pH 7.4), 150 mM NaCl, 1% Triton X/100, 0.3% sodium deoxycholate, 0.1% SDS buffer containing a protease inhibitor cocktail (Sigma-Aldrich, France). After centrifugation at 16,000 *g* for 1 min, the supernatant was incubated with Chromotek GFP-trap agarose (Proteintech, United Kingdom) overnight at 4°C. After centrifugation at 16,000 *g* for 1 min, the supernatant was discarded and the pellet was suspended in 0.5 ml buffer (50 mM Tris (pH 7.4), 150 mM NaCl, 1% Triton X/100, 0.3% sodium deoxycholate, 0.1% SDS), rinsed twice, and analyzed by Heat Shock 70 KDa Protein 5 ELISA assay (#CL-SEC343Mu, Euromedex, France).

**Behavioral tests**

**Object recognition test**

*Familiarization session*: mice were placed individually in the open-field (35 cm x 35 cm x 35 cm) with two identical objects (towers of Lego bricks or Falcon tissue culture flasks filled with sand) positioned 5 cm away from the walls. Animals were left there until they reached the 20-second criterion of total exploration, but no longer than 10 min. Animals that did not meet this criterion were excluded from further studies.

*Test phase*: mice were placed again in the open-field, but this time one of the objects was replaced with the new one (either a tower of Lego bricks or Falcon tissue culture flasks filled with sand). The position of the novel object (left or right) was randomized between each mouse and each group tested. Mice were again left in the open field until they reached the 20-s criterion of total exploration, but no longer than 10 min. After each test experiment, the objects and the open field were cleaned with the odorless veterinary disinfectant to minimize any olfactory cues. The experiments were video-recorded and scored using Eleven Maze (https://elevenmaze.com) software by a trained observer blind to the treatments. To assess animals' performance in the object recognition test, the means of the novel object exploration time were compared with the chance level (10 s, equal exploration of the objects).

HBK-15 was administered *ip* 30 min before the familiarization session or the test phase to verify whether the tested compound affected encoding, or retrieval, respectively (Fig. 2a). MK-801 was injected *ip* 15 min after the administration of the tested compound. Moreover, we investigated how HBK-15 affected the animals' performance in various time intervals – the gap between familiarization and test phase was 15 min, 4 h, and 24 h (encoding process) or 4 h and 24 h (retrieval processes).

To investigate whether the anti-amnesic properties of HBK-15 depend on sigma-1 receptors, the chaperone proteins were blocked using the reference antagonist BD 1047, administered *ip* either 45 min before the familiarization session, or 45 min before the test phase (Fig. 2g). HBK-15 was given 15 min after the sigma-1 receptor antagonist, followed by an *ip* injection of MK-801 15 min later.

**Morris water-maze**

*Acquisition phase (days 1-6):* Animals were placed in a circular pool (height 60 cm, diameter 120 cm; Panlab-Harvard Apparatus, Spain) filled with opaque water at a temperature of 24±1°C, virtually divided into four parts. In one quadrant of the pool, a round platform (diameter 10 cm) was hidden 1 cm below the water surface. Color geometric figures placed near the pool served as visual cues for the swimming animals. Each day, the mouse needed to complete four trials separated by 15-minute time intervals. During each trial, the mouse was placed in different quadrants but never in the one where the platform was located. If the animal did not find the platform within 60 s, it was gently guided to it and left for 15 s. HBK-15 was administered *ip* 30 min before the first trial for six consecutive days, and MK-801 was injected *ip* 15 min later (Figs. 3a, S5a). Vortioxetine (5 mg/kg) and lurasidone (1.25 mg/kg) were administered as reference compounds, based on prior reports of anti-amnesic activity in rodents and patients [4,5]. The whole experiment was video-recorded and analyzed by Eleven Maze (https://elevenmaze.com) software. The following parameters were collected: the latency to enter the platform (i.e., escape latency), the total distance, and the swimming speed.

*Probe test*: 24 h after the last training session, no compounds were administered, and the platform was removed from the pool. Each mouse swam during a 60-s trial, and the following parameters were collected and analyzed: the latency and the covered distance to the target zone (a place where the platform was previously located), the percentage of time spent in the target quarter, and the swimming speed.

**Neurotransmitters level**

**Tissue homogenates**

*Tissue and homogenates preparation:* 30 min after acute administration of the tested compound or saline, naïve or MK-801-treated mice were sacrificed, and their brains were rapidly removed and chilled in an ice-cold saline solution. The hippocampi were dissected, frozen, and stored at −80 °C until assay. On the day of experiments, tissues were thawed on ice and homogenized (1:10 w/v) in buffer containing 50 mM Tris-HCl, 150 mM NaCl, 2 mM EDTA, and 0.32 M sucrose using a bead homogenizer (Bead Rupture elite, Omni International, USA).

*Analytical method:* Concentrations of dopamine (DA), serotonin (5-HT), acetylcholine (ACh), glutamate (Glu), histamine (HIS), and norepinephrine (NE) in the selected brain structures were measured by the liquid chromatography-tandem mass spectrometry method (LC-MS/MS). Homogenates (50 µL) after the addition of 5 µL of IS mixture (deuterated analogs of analyzed compounds at the concentration of 5 µg/mL) were deproteinized with 150 μL of 0.1% formic acid in acetonitrile. Then samples were shaken for 10 min (IKA Vibrax VXR, Germany) and centrifuged for 5 min at 8000 x g (Eppendorf miniSpin centrifuge, Germany). Supernatants were transferred into the autosampler vials. Samples with the analyte concentration above the calibration curve range were additionally diluted with water. Analytes were separated on the XBridge HILIC (2.1 x 150 mm, 3.5 µm, Waters, USA) analytical column using an Exion LC AC HPLC system (Sciex, USA). The injection volume was 5 μL, and the sample was eluted under isocratic conditions at a flow rate of 0.5 mL/min. The mobile phase consisted of 0.1% formic acid in water and 0.1% formic acid in acetonitrile mixed at the ratio of 30/70 (v/v). A QTRAP 4500 (Sciex, USA) tandem mass spectrometer equipped with an electrospray ionization (ESI) source was used for detection. The spectrometer was operated at unit resolution, monitoring the transitions presented in Table S1. Corresponding deuterated internal standards that enable compensation for potential matrix effects were used for analyte quantification. Mass spectrometric parameters were optimized in positive ionization mode by separate infusion of each standard and internal standard solution into the TurboIonSprayTM interface. The most intense product ions were used in the MRM (multiple reaction monitoring) modes to achieve maximum intensity. The following ESI source parameters were selected: ion source temperature was set to 450 °C; curtain gas to 30 psi and collision gas to medium; ionization voltage was set to 5500V. Data acquisition and processing were performed using Analyst version 1.7 software. The calibration curves were constructed by plotting the ratio of the peak area of the studied compound to IS versus drug concentration and generated by weighted (1/x‧x) linear regression analysis. Due to the high endogenous concentrations of analyzed neurotransmitters and availability of stable isotope standards, calibration curves were constructed based on serial dilutions of the calibrators in water. The stock solutions of analyzed neurotransmitters and deuterated internal standards were prepared in water or methanol at a concentration of 1 mg/mL. Working standard solutions (mixture of all analytes) were prepared at the following concentrations: 0.01, 0.025, 0.05, 0.1, 0.25, 0.5, 1, 2.5, 5, and 10 µg/mL. To prepare samples for the calibration curve, 45 μL of water was spiked with 5 μL of standard solution at an appropriate concentration level, 5 µL of IS mixture, and vortexed for 10 s. Calibration curves were linear in the range from 1 to 1000 ng/mL for Ach, 5-HT, and DA; from 2.5 to 1000 ng/mL for HIS; from 10 to 1000 ng/mL for Glu, and from 25 to 1000 ng/mL for NE. Then, concentrations of neurotransmitters in the analyzed samples were calculated per g of brain tissue by applying appropriate dilution factors. Calculated values of accuracy and precision were within the limits set by the FDA guidelines for the validation of bioanalytical methods. No significant matrix effect was observed, and there were no stability-related problems during the routine analysis of the samples.

**Microdialysates**

*Surgery:* Animals were anesthetized with 2.5% isoflurane (5% for induction) and placed in a stereotaxic frame (ASI Instruments, Warren, MI, USA). Analgesia was provided by the administration of lidocaine subcutaneously at the incision site and carprofen (10 mg/kg) subcutaneously in the back. The guide cannulas (MAB 10.8.IC, AgnTho’s AB, Sweden) were implanted over the hippocampus (AP – 1.93 mm; ML + 1.5 mm; DV – 1.8 mm from the bregma; Fig. S2a), according to the stereotaxic atlas of Franklin and Paxinos [6] and fixed in place with two additional anchor screws and dental cement. After surgery, mice were housed in a high-roofed cage with *ad libitum* access to water and food and monitored for recovery (normal eating, drinking, and defecation).

*Microdialysis:* Following a 3-day recovery period, microdialysis probes (MAB 10.8.1.PES with a cut-off off: 6 kD, AgnTho’s AB, Sweden) were connected to the syringe pump (Univentor 864 Syringe Pump, AgnTho’s AB, Sweden) that delivered aCSF composed of (mM) 147 NaCl, 4 KCl, 2.2 CaCl_2,_ and 1.0 MgCl_2_ at a flow rate 1 μl/min. The monitoring of extracellular levels of neurotransmitters has been performed in freely moving animals. After a 2-h stabilization period, a baseline sample was collected for 30 min. Thereafter, HBK-15 at the dose of 2.5 mg/kg was injected *ip,* and three samples were further collected every 30 min (0-30 min, 30-60 min, and 60-90 min post-injection). Placement of dialysis probes was verified post-mortem in coronal sections (Fig. S2b).

*Analytical method:* Concentrations of acetylcholine (ACh) and glutamic acid (Glu) in microdialysis samples from the mouse hippocampus were measured by the liquid chromatography tandem mass spectrometry method (LC-MS/MS). The analytical method employed was similar to the one described above, with some minor modifications. The sample preparation procedure involved the addition of 3 μl of an internal standard (IS) mixture (deuterated internal standards at a concentration of 500 ng/mL) to 30 μl of dialysate. Then, samples were vortex mixed, transferred to autosampler vials, and 5 μl was injected into the LC-MS/MS system. The separation of analytes was carried out using the XBridge HILIC column (2.1 x 150 mm, 3.5 µm, Waters, USA). Since samples had a high salt concentration, a gradient elution method was employed to extend the retention times of the neurotransmitters being analyzed and minimize ion suppression. The initial mobile phase composition consisted of 25 mM ammonium formate (pH = 3.5) in water (A) and acetonitrile (B) in a ratio of 95% B and was pumped for 2 min. Over the next 3 min, the concentration of B was decreased to 60% and maintained for 5 min. Subsequently, B was further reduced to 50% within 1 min and maintained for 3 min. Finally, the mobile phase composition was returned to the initial ratio of 95% B. The entire HPLC operation lasted for 17 min, and the flow rate of the mobile phase was set at 0.3 mL/min. Calibration curves were prepared in the aCSF and were linear in the range from 1 to 1000 ng/mL for Ach and Glu.

**Electrophysiology *ex vivo***

*Hippocampal slices preparation:* The hippocampus was dissected in ice-cold aCSF composed of (mM): NaCl 124; KCl 3; NaH_2_PO_4_ 1.25; NaHCO_3_ 26; MgSO_4_ 1; CaCl_2_ 2; and glucose 10, previously gassed with 95% O_2_ and 5% CO_2_, pH 7.4. Slices (400 μm thick) were cut perpendicularly to the long axis of the hippocampus with a McIlwain tissue chopper and allowed to recover functionally and energetically for 1 h in a resting chamber, filled with the same solution, at room temperature. Slices were transferred to a recording chamber for submerged slices and continuously superfused at 3 ml/min with the gassed bathing solution at 32°C; drugs were added to this superfusion solution in a closed circuit. Recordings were obtained with an Axoclamp 2B amplifier and digitized (Axon Instruments, Foster City, CA). Individual responses were monitored, and averages of six consecutive responses were continuously stored on a personal computer with the LTP software [7].

*Field postsynaptic potentials (fEPSPs) recording*: fEPSPs were recorded through an extracellular microelectrode (4 M NaCl, 2–6 MΩ resistance) placed in the *stratum radiatum* of the CA1 area (Fig. S3). Stimulation (rectangular 0.1 ms pulses, once every 10 s) was delivered through a concentric electrode placed on the Schaffer collateral–commissural fibers, in the *stratum radiatum* near CA3–CA1 border (Fig. S3a). The intensity of the stimulus (180–290 μA) was initially adjusted to obtain a large fEPSP slope with a minimum population spike contamination. Alteration in synaptic transmission induced by drugs was evaluated as the % of the change in the average slope of the fEPSP in relation to the average slope of the fEPSP measured during the 10 min that preceded the addition of drugs, as described previously [7]. Recordings were obtained with an Axoclamp 2B amplifier and digitized (Axon Instruments, Foster City, CA). Individual responses were monitored, and averages of six consecutive responses were continuously stored on a personal computer with the LTP software [7].

*Long-term potentiation (LTP) induction and quantification:* fEPSPs were recorded through an extracellular microelectrode (4 M NaCl, 2–6 MΩ resistance) placed in the *stratum radiatum* of the CA1 area (Fig. S3b). In LTP experiments, stimulation (rectangular 0.1 ms pulses, once every 10 s) was delivered alternatively to two independent pathways through bipolar concentric electrodes placed on Shaffer collateral/commissural fibers in *stratum radiatum*. LTP was induced by a θ-burst protocol consisting of four trains of 100 Hz, and four stimuli, separated by 200 ms (Fig. S3c). The intensity of the stimulus was maintained during the induction protocol. LTP was quantified as the % of the change in the average slope of the fEPSP taken from 60 min after LTP induction in relation to the average slope of the fEPSP measured during the 10 min that preceded the induction of LTP. In each individual experiment, the same LTP-inducing paradigm was delivered to each pathway. To test the effects of the tested drugs upon LTP, each particular drug was added to the superfusing bath at least 15 min before induction of LTP and remained in the bath up to the end of the experiment. In the experiments where both drugs were tested, HBK-15 was added 15 minutes before MK-801 and remained until the end of the experiments.

**Electrophysiology *in vivo***

*Surgery*: Animals (10 male BALB/c mice) were anesthetized with 1-1.5% isoflurane (3-5% for induction) and placed in a stereotaxic frame (SR-5M-HT, Narishige). The body temperature was maintained at 37 ℃ using a feedback temperature controller (50-7221-F, Harvard Bioscience). Animal heads were shaved using electric clippers and cleaned with ethanol (70%) and iodopovidone. Analgesia was provided by the administration of Naropin (0.2%, 0.08 ml) subcutaneously at the incision site and Rimadyl (0.01%, 0.05 ml) subcutaneously in the back. After exposing the skull, multiple bone screws were implanted. A screw over the cerebellum was used as a ground/reference. After craniotomy (AP -2 mm, ML +1.5 mm from bregma), a bipolar wire electrode (130.3 ± 11.8 kΩ at 1 kHz) (AISI 302, 0.1 mm diameter, GoodFellow) was inserted into the hippocampal CA1 (-1.5 mm deep from the cortical surface) and the site was sealed with biocompatible gel (Kwik-Sil, World Precision Instruments). Dental cement was used to secure the electrode and cover the skull surface and screws. After surgery, mice were housed in pairs of two in a high-roofed cage with ad libitum access to water and food.

*in vivo pharmacology and electrophysiology:* Experiments were performed in a plexiglass plastic box (30 cm width × 30 cm depth × 40 cm height). Electrophysiological signals were monitored using an interface board (C3100, RHD USB Interface Board, Intan Technologies) connected to the mouse via a headstage (C3334, RHD 16-channel headstage, Intan Technologies). All signals were acquired at 1 kHz using freely available software (RHX, Intan Technologies). After multiple days of habituation to the recording condition, 2-hour electrophysiological signals were monitored while the animals received one of the following four pharmacological treatments: 1) saline and saline, 2) saline and MK-801 (0.125 mg/kg), 3) HBK-15 (2.5 mg/kg) and saline, and 4) HBK-15 and MK-801. In each treatment, after 15 minutes of baseline recording, the first injection was given intraperitoneally. The second injection was given intraperitoneally on the contralateral side, 15 min after the first injection. The electrophysiological recording continued for 1.5 hours after the second injection. Four treatments were given to each animal in random order over multiple recording sessions. Each treatment was separated by at least a day.

*Histology:* After all pharmacological and electrophysiological experiments, animals were deeply anesthetized with a mixture of pentobarbital and lidocaine and perfused transcardially with phosphate buffer saline (PBS) followed by 4% paraformaldehyde/0.1 M phosphate buffer, pH 7.4. The brains were removed and immersed in the same fixative overnight at 4 ℃ and then immersed in 30% sucrose in PBS for at least 2 days. The brains were cut into coronal sections with a sliding microtome (SM2010R, Leica) with a 100 µm thickness. To determine the electrode position, the sections were stained with DAPI (1:1000, Sigma-Aldrich) and cover-slipped with an antifade solution (fluoromount-G, ThermoFisher Scientific). Images were taken under the epifluorescence microscope (Eclipse E600, Nikon, Fig. S4).

*Data analysis:* All data analysis was performed offline using custom-written scripts (MATLAB R2022a, MathWorks). To compute theta and gamma power, hippocampal local field potentials (LFPs) were taken from one of two channels, and a spectrogram was computed with a 10-s window and 5-s overlap. In each recording, the power of theta (4-10 Hz) and gamma (30-45 Hz) was calculated and normalized by the total power at ≤40 Hz. Using baseline signals between 5 and 15 min from the beginning of each recording, the Z-score of theta and gamma power was computed. The effect of treatments was assessed by calculating the average Z-scored power of theta and gamma oscillations between 30 and 60 min after the second injection.

To estimate the phase-amplitude coupling (PAC) between theta and gamma oscillations, LFPs were bandpass-filtered at 4-10 Hz and 30-45 Hz as theta and gamma oscillations, respectively, using the third-order Butterworth filter. The instantaneous phase of theta oscillations was estimated as the angle of the Hilbert-transformed signals. The amplitude envelope of gamma oscillations was calculated as the absolute value of the analytical signals after the Hilbert transformation. The mean amplitude of gamma oscillations at a certain phase of theta oscillations was computed in every 30-s window and 45° phase bin. The modulation index was defined as the ratio between the maximum and minimum mean amplitudes in each time window. Similar to the frequency band power described above, the Z-score of the modulation index was calculated across time windows using baseline values between 5 and 15 min from the beginning of each recording. The effect of treatments was assessed by computing the average normalized modulation index between 30 and 60 min after the second injection.

**Statistical analysis**

Results are presented as mean ± standard deviation (SD) or standard error of mean (SEM) (parametric analysis) or median and interquartile range (IQR) (non-parametric analysis).  For behavioral studies, SD was used to reflect individual variability. In contrast, for electrophysiological and IC_50_ studies, we used SEM to emphasize the precision of the mean and ensure accurate representation of experimental conditions. The data set's normality and homogeneity were determined using the Shapiro-Wilk and Brown-Forsythe tests, respectively. The comparisons between groups were performed by one-way ANOVA, repeated measures one-way ANOVA or repeated measures two-way ANOVA, followed by appropriate *post hoc* tests: Dunnett's (when comparing means from several experimental groups against a control group mean), Tukey’s (when comparing means between all groups) and Bonferroni (when comparing the means of the preselected pairs of experimental groups). When the assumption of normal distribution was not met, we used the non-parametric Kruskal-Wallis analysis with Dunn's *post hoc* test. When the homogeneity of variance was violated, we performed the Welch ANOVA test, followed by Dunnett's T3 test. Additionally, we applied the Greenhouse-Geisser correction when the assumption of sphericity was violated for repeated measures ANOVA. To assess animals' performance in the object recognition test, the means of the novel object exploration time were compared with the chance level (10 s, equal exploration of the objects) using a one-sample t-test. In all tests, *p*<0.05 was considered significant. The statistical analyses were performed using GraphPad software 9.5.0 (GraphPad Software, Inc., La Jolla, CA 92037, USA) or MATLAB (R2022a, MathWorks).

References

[1] Hayashi T, Su T-P. Sigma-1 receptor chaperones at the ER-mitochondrion interface regulate Ca(2+)  signaling and cell survival. Cell 2007;131:596–610. https://doi.org/10.1016/j.cell.2007.08.036.

[2] Crouzier L, Meunier J, Carles A, Morilleau A, Vrigneau C, Schmitt M, et al. Convolamine, a tropane alkaloid extracted from Convolvulus plauricalis, is a potent sigma-1 receptor-positive modulator with cognitive and neuroprotective properties. Phytotherapy Research 2024;38:694–712. https://doi.org/10.1002/PTR.8068.

[3] García-Pupo L, Crouzier L, Bencomo-Martínez A, Meunier J, Morilleau A, Delprat B, et al. Amylovis-201 is a new dual-target ligand, acting as an anti-amyloidogenic compound and a potent agonist of the σ1 chaperone protein. Acta Pharm Sin B 2024;14:4345. https://doi.org/10.1016/J.APSB.2024.06.013.

[4] Bozkurt NM, Unal G. Vortioxetine improved negative and cognitive symptoms of schizophrenia in subchronic MK-801 model in rats. Behavioural Brain Research 2023;444:114365. https://doi.org/10.1016/j.bbr.2023.114365.

[5] Harvey PD, Ogasa M, Siu C, Loebel A. Lurasidone Effects on Cognition and Functional Capacity in Schizophrenia. Clinical Neuropsychopharmacology and Therapeutics 2016;7:11–9. https://doi.org/10.5234/cnpt.7.11.

[6] Paxinos G, Franklin K. Paxinos and Franklin’s the Mouse Brain in Stereotaxic Coordinates. 5th Editio. London: Academic Press; 2019.

[7] Fonseca-Gomes J, Costa-Coelho T, Ferreira-Manso M, Inteiro-Oliveira S, Vaz SH, Alemãn-Serrano N, et al. A small TAT-TrkB peptide prevents BDNF receptor cleavage and restores synaptic physiology in Alzheimer disease. Molecular Therapy 2024;32:3372–401. https://doi.org/10.1016/j.ymthe.2024.08.022.

[8] Ganapathy ME, Prasad PD, Huang W, Seth P, Leibach FH, Ganapathy V. Molecular and ligand-binding characterization of the sigma-receptor in the Jurkat  human T lymphocyte cell line. J Pharmacol Exp Ther 1999;289:251–60.

[9] Monaghan DT, Cotman CW. The distribution of [3H]kainic acid binding sites in rat CNS as determined by  autoradiography. Brain Res 1982;252:91–100. https://doi.org/10.1016/0006-8993(82)90981-7.

[10] Honoré T, Lauridsen J, Krogsgaard-Larsen P. The binding of [3H]AMPA, a structural analogue of glutamic acid, to rat brain  membranes. J Neurochem 1982;38:173–8. https://doi.org/10.1111/j.1471-4159.1982.tb10868.x.

[11] Sills MA, Fagg G, Pozza M, Angst C, Brundish DE, Hurt SD, et al. [3H]CGP 39653: a new N-methyl-D-aspartate antagonist radioligand with low nanomolar  affinity in rat brain. Eur J Pharmacol 1991;192:19–24. https://doi.org/10.1016/0014-2999(91)90063-v.

[12] Siegel BW, Sreekrishna K, Baron BM. Binding of the radiolabeled glycine site antagonist [3H]MDL 105,519 to homomeric  NMDA-NR1a receptors. Eur J Pharmacol 1996;312:357–65. https://doi.org/10.1016/0014-2999(96)00477-3.

[13] Javitt DC, Zukin SR. Interaction of [3H]MK-801 with multiple states of the N-methyl-D-aspartate receptor  complex of rat brain. Proc Natl Acad Sci U S A 1989;86:740–4. https://doi.org/10.1073/pnas.86.2.740.

[14] Schoemaker H, Allen J, Langer SZ. Binding of [3H]ifenprodil, a novel NMDA antagonist, to a polyamine-sensitive site in  the rat cerebral cortex. Eur J Pharmacol 1990;176:249–50. https://doi.org/10.1016/0014-2999(90)90539-i.

[15] Mutel V, Ellis GJ, Adam G, Chaboz S, Nilly A, Messer J, et al. Characterization of [(3)H]Quisqualate binding to recombinant rat metabotropic  glutamate 1a and 5a receptors and to rat and human brain sections. J Neurochem 2000;75:2590–601. https://doi.org/10.1046/j.1471-4159.2000.0752590.x.

[16] Johnson BG, Wright RA, Arnold MB, Wheeler WJ, Ornstein PL, Schoepp DD. [3H]-LY341495 as a novel antagonist radioligand for group II metabotropic glutamate  (mGlu) receptors: characterization of binding to membranes of mGlu receptor subtype expressing cells. Neuropharmacology 1999;38:1519–29. https://doi.org/10.1016/s0028-3908(99)00053-2.

[17] Wright RA, Arnold MB, Wheeler WJ, Ornstein PL, Schoepp DD. Binding of [3H](2S,1’S,2’S)-2-(9-xanthylmethyl)-2-(2’-carboxycyclopropyl) glycine  ([3H]LY341495) to cell membranes expressing recombinant human group III metabotropic glutamate receptor subtypes. Naunyn Schmiedebergs Arch Pharmacol 2000;362:546–54. https://doi.org/10.1007/s002100000305.

[18] Reynolds IJ, Snowman AM, Snyder SH. (-)-[3H] desmethoxyverapamil labels multiple calcium channel modulator receptors in  brain and skeletal muscle membranes: differentiation by temperature and dihydropyridines. J Pharmacol Exp Ther 1986;237:731–8.

[19] Gee NS, Brown JP, Dissanayake VU, Offord J, Thurlow R, Woodruff GN. The novel anticonvulsant drug, gabapentin (Neurontin), binds to the alpha2delta  subunit of a calcium channel. J Biol Chem 1996;271:5768–76. https://doi.org/10.1074/jbc.271.10.5768.

[20] Hope AG, Peters JA, Brown AM, Lambert JJ, Blackburn TP. Characterization of a human 5-hydroxytryptamine3 receptor type A (h5-HT3R-AS)  subunit stably expressed in HEK 293 cells. Br J Pharmacol 1996;118:1237–45. https://doi.org/10.1111/j.1476-5381.1996.tb15529.x.

**Supplementary Figures**

**Fig. S1. Chemical structure of 1-[(2-chloro-6-methylphenoxy)ethoxyethyl]-4-(2-methoxyphenyl)piperazine hydrochloride (HBK-15)**

**
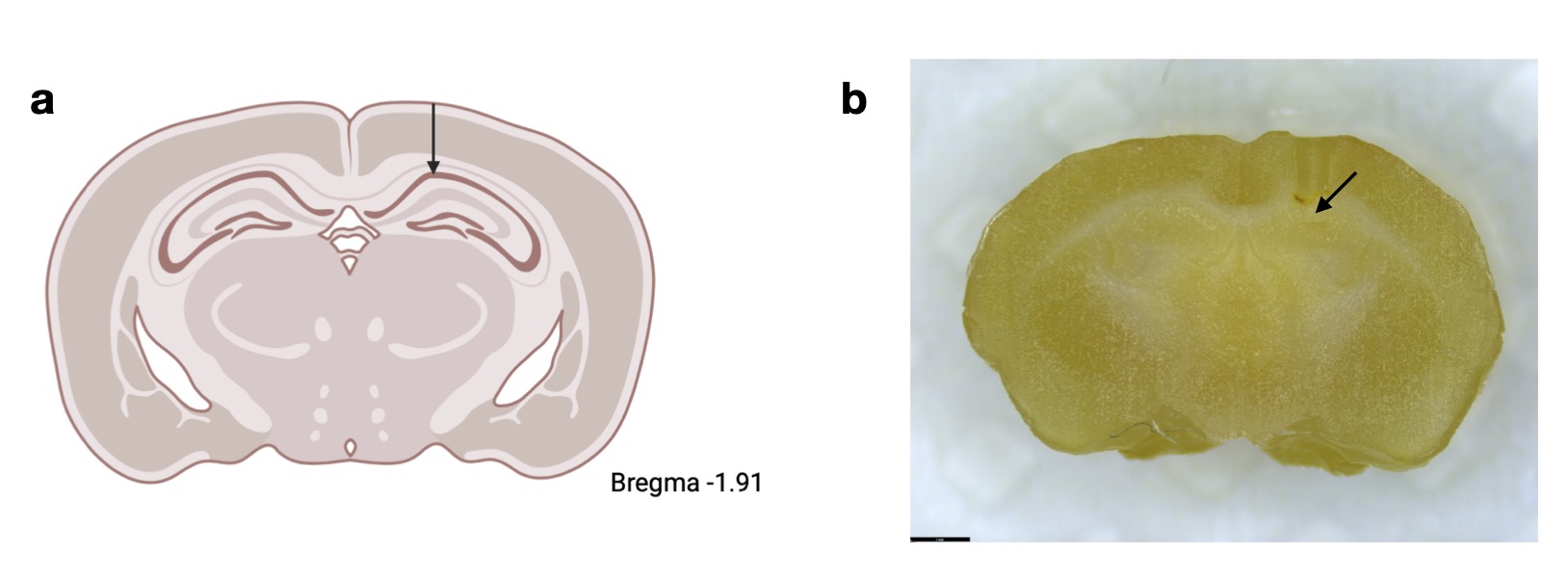
**

**Fig. S2. Microdialysis cannula positions.** a: Arrow indicates the representative placement of the cannula. b: Coronal section of brain showing the cannula placement (scale bar – 1 mm).


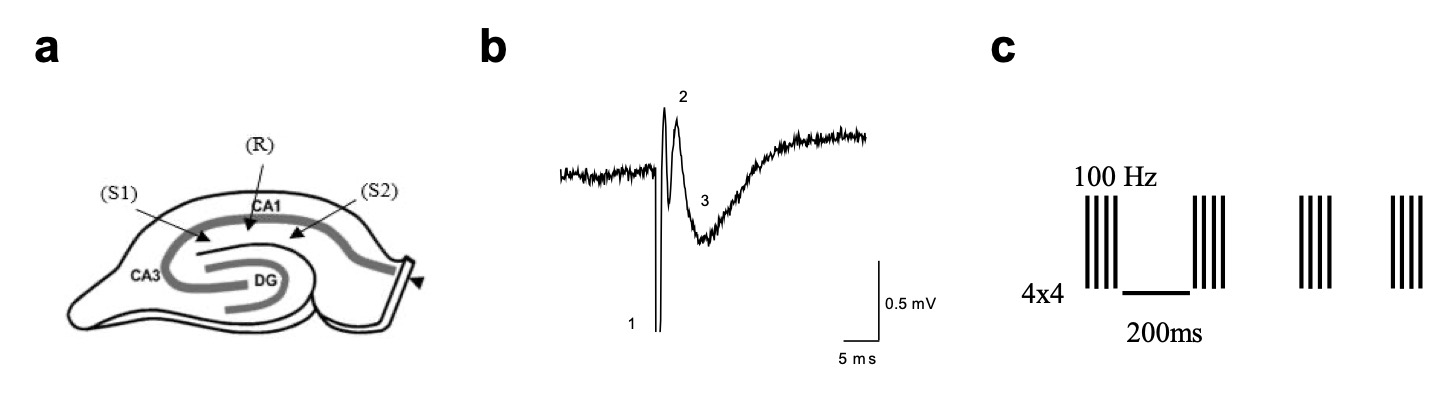


**Fig. S3. Extracellular recordings in hippocampal slices.** a: Schematic representation of a hippocampal transverse slice preparation showing the recording configuration used to obtain extracellular responses in the CA1 dendritic layer (*stratum radiatum*) evoked by stimulation of two separate sets of the Schaffer pathway (S1 and S2). b: Examples of representative traces obtained after stimulation are composed of the stimulus artifact (1), followed by the pre-synaptic volley (2), and the field excitatory post-synaptic potentials (fEPSP) (3). c: A schematic representation of the stimulation paradigms used in plasticity experiments.


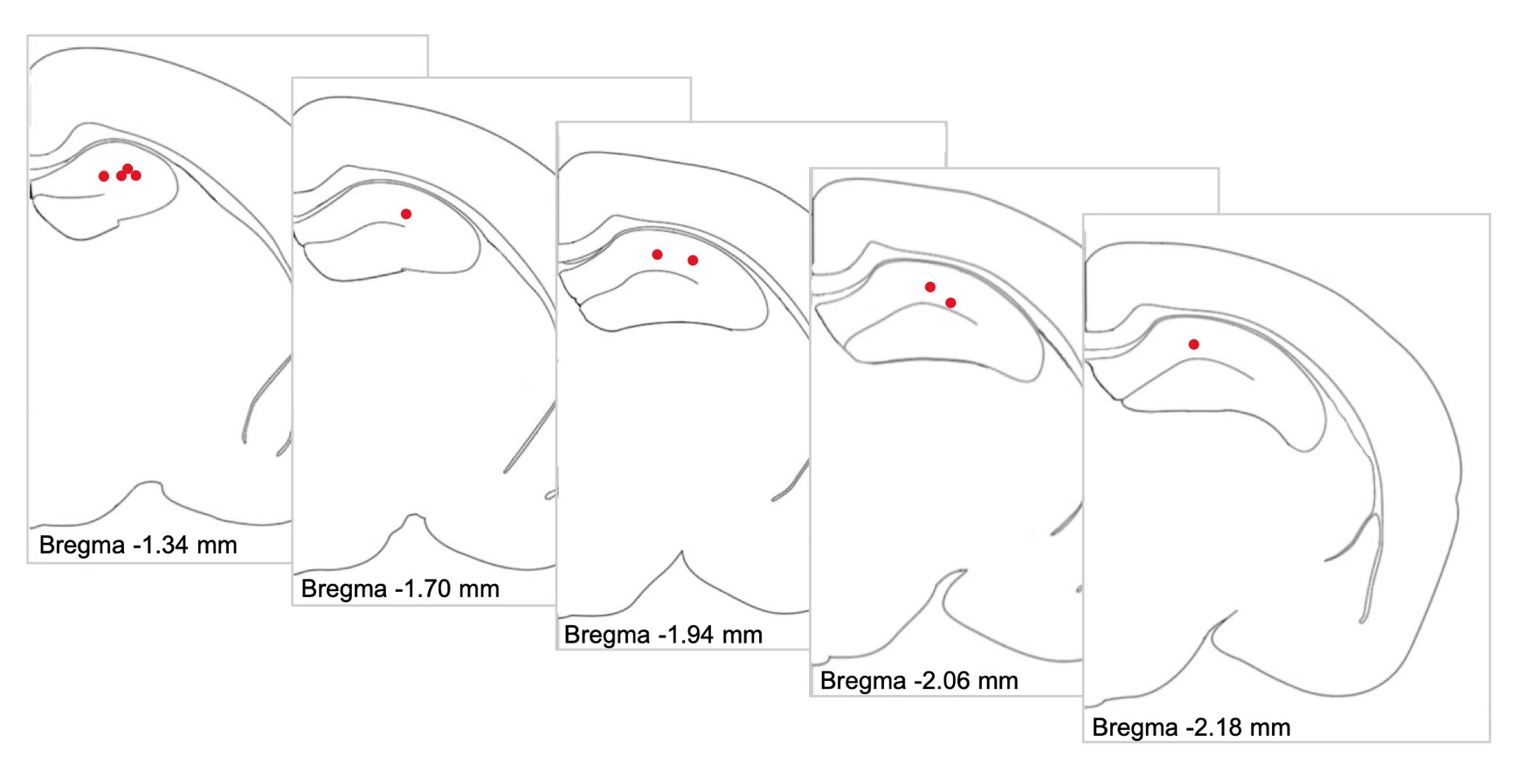


**Fig. S4. Electrode positions.** Red dots indicate the electrode tip position based on histological analysis.

**Fig. S5.** **HBK-15 reversed MK-801-induced spatial learning and memory deficits in the Morris water maze. a:** Experimental scheme: HBK-15 and MK-801 were administered intraperitoneally *(ip)* 30 min before the first trial for six consecutive days, and on the seventh day of the experiment, no treatment was administered. **b,c:** The effect of HBK-15 on the covered distance (**b**) and animals' speed (**c**) in the acquisition phase. The data are presented as means of four trials ± SD. Statistical analysis: two-way ANOVA for repeated measurements with Greenhouse-Geisser correction (Bonferroni *post hoc*), **p* <0.05, ***p* <0.01, ****p* <0.001, *****p* <0.0001; distance - time: *F*(4.266,345.5) = 151, *p* <0.0001; treatment: *F*(8,81) = 8.524, *p* <0.0001; interaction: *F*(40,405) = 3.019, *p* <0.0001; swimming speed - time: *F*(2.937,237.9) = 8.925, *p* <0.0001; treatment: *F*(8,81) = 3.958, *p* <0.001; interaction: *F*(40,405) = 1.667, *p* = 0.0083; n = 10 mice per group. **d,e,f:** The influence of HBK-15 on the distance covered to find the target zone (**d**) and animals' speed (**e**) in the probe test (seventh day of the experiment). The data are presented as means ± SD (**d**) or medians with IQR (**e**). Statistical analysis: Welch ANOVA (Dunnett's T3 *post hoc*; **d**) or Kruskal-Wallis test (Dunn *post hoc* **e**), distance: *W*(8.0,31.13) = 16.42, *p* <0.0001, speed – *H*(9,86) = 17.64, *p* = 0.024; n = 8-10 mice per group

**Supplementary tables**

**Table S1. Summary of receptor-binding assays for HBK-15 performed by radioligand binding.**

| **Molecular target** | **Source** | **Ligand (dose)** | **Non-specific ligand (dose)** | **Reference** |
| --- | --- | --- | --- | --- |
| sigma 1 | Human endogenous Jurkat cells | [^3^H]  (+)pentazocine  (15 nM) | Haloperidol  (10 µM) | [8] |
| sigma 2 | Human endogenous Jurkat cells | [^3^H] DTG (25 nM)  (+1µM (+)  Pentazocine) | Haloperidol (10 µM) | [8] |
| KA | Rat cerebral cortex | [^3^H]kainic acid  (5 nM) | L-glutamate  (1 mM) | [9] |
| AMPA | Wistar rat cerebral cortex | [^3^H] AMPA  (5 nM) | L-Glutamic acid (1 mM) | [10] |
| NMDA (Glu) | Wistar rat cerebral cortex | [^3^H] CGP-39653  (2 nM) | L-Glutamic acid (1mM) | [11] |
| NMDA (Gly) | Wistar rat cerebral cortex | [^3^H] MDL 105,519 (0.33 nM) | MDL 105,519  (10 µM) | [12] |
| NMDA  (MK-801) | Wistar rat brain (minus cerebellum) | [^3^H] MK-801  (5 nM) | (+)-MK-801  (10 µM) | [13] |
| NMDA (PA) | Wistar rat cerebral cortex | [^3^H] Ifenprodil  (2 nM) | Ifenprodil  (10 µM) | [14] |
| mGluR1 | Rat cerebellum | [^3^H] quisqualate  (40 nM) | L-Glutamate  (1 mM) | [15] |
| mGluR2 | Human recombinant Chem-1 cells | [^3^H] LY341495  (2 nM) | LY-354740  (5 µM) | [16,17] |
| mGluR5 | Human recombinant CHO-K1 cells | [^3^H] Quisqualic acid (0.03 µM) | L-Glutamic acid (1 mM) | [15] |
| Cav1.2 (phenylalkylamine) | Wistar rat cerebral cortex | [^3^H] (-)-Desmethoxyverapamil (D-  888; 5 nM) | Methoxyverapamil (D-600; 10 µM) | [18] |
| Cav2.2 (𝜔-conotoxin) | Human recombinant CHO cells | [^125^I] ω-Conotoxin GVIA  (20 pM) | ω-Conotoxin GVIA  (1 µM) | [19] |
| Cav2.2 (gabapentin) | Human recombinant CHO cells | [^3^H] Gabapentin  (5 nM) | Gabapentin  (10 µM) | [19] |
| 5-HT_3_ | Human recombinant CHO cells | [^3^H] BRL 43694  (0.5 nM) | MDL 72222  (10 µM) | [20] |

Binding studies were performed commercially in Eurofins Laboratories (Poitiers, France). The binding sites for N-methyl-D-aspartate (NMDA) receptors are presented in brackets. Glu – glutamate, Gly – glycine, PA – polyamines, KA - kainate, AMPA - α-amino-3-hydroxy-5-methyl-4-isoxazolepropionic acid

**Table S2. Monitored transitions and ion path parameters of analyzed neurotransmitters and deutereted internal standards.**

| Analyte | DA | DAd4 | 5-HT | 5-HTd4 | HIS | HISd4 | NE | NEd6 | Glu | Glud5 | ACh | AChd9 |
| --- | --- | --- | --- | --- | --- | --- | --- | --- | --- | --- | --- | --- |
| Monitored transitions | 154/137 | 158/141 | 177/160 | 181/164 | 112/95 | 116/99 | 170/152 | 176/158 | 148/84 | 153/88 | 146/87 | 155/87 |
| Declustering potential (DP) | 46 | 56 | 131 | 136 | 66 | 6 | 136 | 41 | 26 | 1 | 1 | 1 |
| Collision cell exit potential (CXP) | 10 | 10 | 12 | 12 | 8 | 8 | 10 | 10 | 8 | 8 | 8 | 8 |
| Collision Energy (CE) | 15 | 15 | 11 | 15 | 21 | 25 | 11 | 13 | 21 | 23 | 19 | 19 |

DA – dopamine, 5-HT – serotonin, HIS – histamine, NE – norepinephrine, Glu – glutamic acid, ACh - acetylcholine

**Table S3. The effect of the single administration of HBK-15 on the histamine, serotonin, acetylcholine, noradrenaline, dopamine, and glutamate levels in the hippocampus homogenates of naïve mice.**

| **Treatment** | **Dose [mg/kg]** | **Level [ng/g] or [μg/g] (glutamate)** | | | | | | | | | | | |
| --- | --- | --- | --- | --- | --- | --- | --- | --- | --- | --- | --- | --- | --- |
|  |  | **histamine** | | | **serotonin** | | | **acetylcholine** | **noradrenaline** | **glutamate** | | | **dopamine** |
| saline | - | 507.7 | ± | 176.0 | 360.6 | ± | 97.54 | 1153 (156.0) | 1337.0 (939.1) | 2145 | ± | 786.7 | < LOQ (20 ng/g) |
| HBK-15 | 0.3 | 420.9 | ± | 89.36 | 273.1 | ± | 82.82 | 1049 (291.6) | 1003.0 (649.4) | 1793 | ± | 431.0 |  |
|  | 0.625 | 510.8 | ± | 160.7 | 283.9 | ± | 72.19 | 1121 (235.0) | 1195.0 (747.3) | 2220 | ± | 348.5 |  |
|  | 1.25 | 616.0 | ± | 96.16 | 298.9 | ± | 64.93 | 1199 (200.0) | 1154.0 (671.0) | 2131 | ± | 332.4 |  |
|  | 2.5 | 492.5 | ± | 108.9 | 324.1 | ± | 74.72 | 1115 (186.0) | 1055.0 (983.4) | 1844 | ± | 189.9 |  |
|  | 5 | 515.1 | ± | 149.2 | 336.7 | ± | 76.93 | 1092 (295.0) | 878.2 (1893.1) | 1907 | ± | 326.2 |  |

30 min after a single administration of HBK-15 or saline (control group), mice were sacrificed, and then the hippocampi were isolated. The level of selected neurotransmitters was determined using high-performance liquid chromatography combined with mass spectrometry. Values ​​are expressed as means ± SD (histamine, serotonin, glutamate) or medians and IQR (acetylcholine, noradrenaline). Statistical analysis: histamine, serotonin: one-way ANOVA (Bonferroni *post hoc*); acetylcholine, noradrenaline: Kruskal-Wallis test (Dunn *post hoc*); glutamate: Welch ANOVA (Dunnett's T3 *post hoc*); serotonin: *F*(5,54) = 1.798, *p* = 0.8842, noradrenaline: *H*(5,59) = 2.902, *p* = 0.715, histamine: *F*(5,54) = 2.165, *p* = 0.0716, dopamine: concentration below LOQ, glutamate: *W*(5,23.18) = 2.502, *p* = 0.0596, acetylcholine: *H*(5,60) = 2.797, *p* = 0.7312; n = 8-10 mice per group

**Table S4. The effect of the single administration of HBK-15 on the acetylcholine and glutamate levels in the microdialysates from the hippocampus of naïve mice.**

| **Time post-injection of HBK-15 at a dose of 2.5 mg/kg** | **Level [ng/ml]** | | | | | |
| --- | --- | --- | --- | --- | --- | --- |
|  | **acetylcholine** | | | **glutamate** | | |
| Baseline (-30-0 min) | 4.417 | ± | 1.318 | 80.68 | ± | 53.40 |
| 0-30 min | 4.369 | ± | 1.343 | 89.88 | ± | 72.98 |
| 30-60 min | 4.330 | ± | 1.432 | 98.01 | ± | 66.89 |
| 60-90 min | 4.146 | ± | 1.212 | 88.13 | ± | 66.26 |

*In vivo* microdialysis was used to analyze extracellular acetylcholine and glutamate levels in the hippocampus of naïve, freely moving mice. Four samples were collected every 30 min after the washout period of 2 h (baseline, 30 min, 60 min, and 90 min post-injection of HBK-15. The level of selected neurotransmitters was determined using high-performance liquid chromatography combined with mass spectrometry. Values ​​are expressed as means ± SD. Statistical analysis: one-way repeated measures ANOVA (Dunnett’s *post hoc*), glutamate: *F*(2.346,18.77) = 0.1948, *p* = 0.8561; acetylcholine: *F*(1.495,13.45) = 0.6435, *p* = 0.4973; n = 9-10 mice per group

**Table S5. The effect of the single administration of HBK-15 on the histamine, serotonin, noradrenaline, and dopamine levels in the hippocampus homogenates of MK-801-treated mice.**

| **Treatment** | | **Dose [mg/kg]** | **Level [ng/g]** | | | | | |
| --- | --- | --- | --- | --- | --- | --- | --- | --- |
|  |  |  | **histamine** | **serotonin** | | | **noradrenaline** | **dopamine** |
|  | saline | - | 157.8 (63.9) | 14.86 | ± | 3.57 | 50.91 (19.94) | < LOQ (20 ng/g) |
| MK-801 (0.125 mg/kg) | saline | - | 151.2 (28.5) | 12.15 | ± | 2.362 | 45.57 (24.44) |  |
|  | HBK-15 | 0.3 | 145.6 (47.9) | 11.98 | ± | 3.422 | 42.71 (26.11) |  |
|  |  | 0.625 | 169.5 (35.1) | 9.026 | ± | 2.16 | 72.00 (40.75) |  |
|  |  | 1.25 | 130.4 (79.1) | 14.12 | ± | 4.684 | 47.83 (68.98) |  |
|  |  | 2.5 | 145.6 (51.9) | 8.651 | ± | 2.06 | 60.23 (42.25) |  |
|  |  | 5 | 187.5 (62.7) | 14.87 | ± | 2.726 | 35.05 (30.32) |  |

Mice were administered a single dose of HBK-15 or saline (control group), followed 15 min later by an injection of MK-801 or saline. 15 min after the second injection, the mice were sacrificed, and their hippocampi were isolated. The level of selected neurotransmitters was determined using high-performance liquid chromatography combined with mass spectrometry. Values are expressed as means ± SD (serotonin) or medians and IQR (histamine, noradrenaline). Statistical analysis: serotonin: one-way ANOVA (Bonferroni *post hoc*); histamine, noradrenaline: Kruskal-Wallis test (Dunn *post hoc*); serotonin: *F*(6,62) = 6.885, *p* < 0.0001, noradrenaline: *H*(6,70) = 14.44, *p* = 0.0251, histamine: *F*(6,70) = 10.44, *p* = 0.1072, dopamine: concentration below LOQ, n = 9-10 mice per group
